# Identification of loci and candidate gene *GmSPX-RING1* responsible for phosphorus efficiency in soybean via genome-wide association analysis

**DOI:** 10.1186/s12864-020-07143-3

**Published:** 2020-10-19

**Authors:** Wenkai Du, Lihua Ning, Yongshun Liu, Shixi Zhang, Yuming Yang, Qing Wang, Shengqian Chao, Hui Yang, Fang Huang, Hao Cheng, Deyue Yu

**Affiliations:** 1 National Center for Soybean Improvement, National Key Laboratory of Crop Genetics and Germplasm Enhancement, Jiangsu Collaborative Innovation Center for Modern Crop Production, Nanjing Agricultural University, Nanjing, 210095 China; 2 Institute of Crop Germplasm and Biotechnology, Provincial Key Laboratory of Agrobiology, Jiangsu Academy of Agricultural Sciences, Nanjing, 210014 China; 3 School of Life Sciences, Guangzhou University, Guangzhou, 510006 China

**Keywords:** Soybean, GWAS, P efficiency, *GmSPX-RING1*, P concentration

## Abstract

**Background:**

Phosphorus (P) is an essential element in maintaining high biomass and yield in crops. Soybean [*Glycine max* (L.) Merr.] requires a large amount of P during growth and development. Improvement of P efficiency and identification of P efficiency genes are important strategies for increasing soybean yield.

**Results:**

Genome-wide association analysis (GWAS) with NJAU 355 K SoySNP array was performed to identify single nucleotide polymorphisms (SNPs) significantly associated with three shoot P efficiency-related traits of a natural population of 211 cultivated soybeans and relative values of these traits under normal P (+P) condition and P deficiency (−P) condition. A total of 155 SNPs were identified significantly associated with P efficiency-related traits. SNPs that were significantly associated with shoot dry weight formed a SNP cluster on chromosome 11, while SNPs that were significantly associated with shoot P concentration formed a SNP cluster on chromosome 10. Thirteen haplotypes were identified based on 12 SNPs, and Hap9 was considered as the optimal haplotype. Four SNPs (AX-93636685, AX-93636692, AX-93932863, and AX-93932874) located on chromosome 10 were identified to be significantly associated with shoot P concentration under +P condition in two hydroponic experiments. Among these four SNPs, two of them (AX-93636685 and AX-93932874) were also significantly associated with the relative values of shoot P concentration under two P conditions. One SNP AX-93932874 was detected within 5′-untranslated region of *Glyma.10 g018800*, which contained SPX and RING domains and was named as *GmSPX-RING1.* Furthermore, the function research of *GmSPX-RING1* was carried out in soybean hairy root transformation. Compared with their respective controls, P concentration in *GmSPX-RING1* overexpressing transgenic hairy roots was significantly reduced by 32.75% under +P condition; In contrast, P concentration in RNA interference of *GmSPX-RING1* transgenic hairy roots was increased by 38.90 and 14.51% under +P and -P conditions, respectively.

**Conclusions:**

This study shows that the candidate gene *GmSPX-RING1* affects soybean phosphorus efficiency by negatively regulating soybean phosphorus concentration in soybean hairy roots. The SNPs and candidate genes identified should be potential for improvement of P efficiency in future soybean breeding programs.

## Background

Phosphorus (P) is one of the essential mineral elements in plant growth and reproduction [1] and it is involved in many metabolic processes such as energy and cell membrane formation, nucleic acid synthesis, photosynthesis and respiration. However, nearly half of the world’s arable land is P deficient [2]. P deficiency severely restricts crop production, especially oil crops such as soybean [*Glycine max* (L.) Merr.]. From 1961 to 2013, the total amount of agricultural phosphate fertilizer used in the world increased from 4.6 to 17.5 million tons per year. And the use of phosphate fertilizer per unit cropland area increased by about 3 times in the same period [3]. Applying phosphate fertilizer increases crop yield, but only orthophosphate ions (H_2_PO_4_^−^ and HPO_4_^2−^) can be directly absorbed and utilized by plants [4] and 80–90% of phosphate fertilizer applied to the soil is adsorbed by microorganisms, or forms insoluble chelate with metal ions [5]. The insoluble phosphate fertilizer fixed in the soil is washed by rainwater, resulting in eutrophication of the water body. Therefore, improving crop P efficiency is one of the effective strategies to improve food production and reduce water eutrophication.

P efficiency usually includes P acquisition efficiency (PAE) and P use efficiency (PUE). PAE refers to the ability of crops to uptake P, while PUE refers to the ability of biomass or yield produced using the acquired P [6]. In order to improve P efficiency in crops, it is important to explore the molecular mechanisms of genetic variation in different accessions. Previous studies show that plants have formed a series of adaptation mechanisms to improve P efficiency such as changing of root system architecture [7], secreting of organic acids [8] and purple acid phosphatases [9], enhancing expression of phosphate transporters [10], and utilizing arbuscular mycorrhizal symbiosis [11] etc. Many genes related to P efficiency have been identified, such as acid phosphatase [12], protein kinase [13] and E3 ubiquitin ligase [14] etc. In particular, proteins containing SPX domain (SYG1/PHO81/XPR1) that are involved in regulation of P efficiency have been widely studied in plants. There are 4, 6 and 10 SPX family members in *Arabidopsis* (*Arabidopsis thaliana*) [15], rice (*Oryza sativa*) [16] and soybean [*Glycine max* (L.) Merr.] [17], respectively. The proteins containing SPX domain in plants are divided into 4 families, named as SPX, SPX-EXS, SPX-MFS and SPX-RING families [18]. The SPX-RING family contains two domains: SPX at the N-terminus and RING at the C-terminus, and RING domain is responsible for putative ubiquitin E3 ligase activity [19].

To date, only one SPX-RING family gene, *Nitrogen-Limited Adaptation* (*NLA*), has been identified in *Arabidopsis*. *Arabidopsis nla* mutant was identified to be unable to accumulate anthocyanin and showed accelerated senescence compared with WT in the absence of nitrogen [14, 20]. Further studies indicated that *Arabidopsis nla* mutant exhibited premature senescence at low nitrogen due to P toxicity [21]. *Arabidopsis nla* mutant contained around twofold higher content of P than that of the wild-type plants in both shoots and roots [22]. The homologous gene *OsNLA1* in rice was also identified. P concentration in *osnla1* leaves significantly increased and the change was independent of nitrogen [19].

Soybean is one of the major food crops and is the main source of vegetable protein and oil [23]. A large amount of phosphate fertilizer is needed in soybean production, but application of phosphate fertilizer and the amount of phosphate fertilizer in different periods has different effects on soybean yield. Recent study has shown that when the total fertilization amount is the same and phosphate fertilizer is used as top dressing which would be mostly used in the growth process of crops (nitrogen and potassium are top dressings as controls), the number of soybean pods and 100-grain weight increase, and the yield also increases significantly [24]. P deficiency in soybean seedling stage (V3-R1) could reduce the node number and kernel number, which could significantly reduce the yield [25]. Hence, it is important to identify P efficiency genes and understand their molecular mechanisms in soybean seedling to increase soybean yield.

Quantitative trait loci (QTL) mapping [26] and genome-wide association study (GWAS) [27] are the most commonly used and effective methods for identifying genes in forward genetics. So far, many QTLs associated with P efficiency in seedling stage have been identified. Three QTLs related to fresh shoot weight were mapped on linkage group F2; and two QTLs related to P content in leaf were mapped on linkage group F1 [28]. Three clusters of QTLs mapped on linkage group B2–1, D1b + W and G were related to dry weight, PAE and PUE [29]. Some SNPs that were significantly associated with P efficiency had also been discovered by GWAS. The conditional phenotypes of five shoot related traits were identified to be significantly associated with three SNPs on chromosome 3 [30]. *GmACP1* (*Acid phosphatase 1*) identified by combining QTL mapping and GWAS showed significantly increased expression and higher acid phosphatase activity after P starvation, thereby improving soybean P efficiency [31]. *GmHAD1* (*HAD-like acid phosphatase 1*) identified by QTL mapping showed increased P efficiency in soybean hairy roots and higher biomass in transgenic *Arabidopsis* [32]. These studies suggested that P efficiency-related traits were quantitative trait loci controlled by multiple genes, and many genes related to P efficiency needed to be further identified.

In this study, GWAS with NJAU 355 K SoySNP array [33] was performed to identify P efficiency related novel loci, favorable haplotypes, and genes in soybean across two hydroponic experiments (E1 and E2). A novel SNP (AX-93932874) significantly associated with P efficiency-related trait was detected within 5′-untranslated region of *Glyma.10 g018800*. In addition, the results of gene expression analyses, bioinformatics, subcellular localization and soybean hairy root transformation indicated that *GmSPX-RING1* containing SPX and RING domains had a negative regulatory effect on P concentration in soybean hairy roots. These results will contribute to the breeding of higher P efficiency soybean accessions.

## Results

### Significant variation and correlation coefficients of three P efficiency-related traits in soybean accessions

In order to study the P efficiency in different soybean accessions, three P efficiency-related traits including shoot dry weight (SDW), shoot P concentration (SP) and shoot P accumulation (SPA) were measured in a natural population containing 211 cultivated soybean accessions. In addition, the relative values of these three traits including the ratio of shoot dry weight under P deficiency (−P, 0.005 mM KH_2_PO_4_) condition to normal P (+P, 0.5 mM KH_2_PO_4_) condition (SDWR), the ratio of shoot P concentration under -P condition to +P condition (SPR) and the ratio of shoot P accumulation under -P condition to +P condition (SPAR) were also analyzed. The mean value, standard deviation, coefficient of variation, range and broad-sense heritability (*h*^*2*^) of three traits under two P conditions and relative values of these traits in two hydroponic experiments (E1 and E2) were analyzed (Table 1, Additional file 1: Figure S1). In 211 cultivated soybean accessions, mean value of three P efficiency-related traits (SDW, SP and SPA) under -P condition were lower than those under +P condition in E1 and E2 respectively. The phenotypic values for this natural population ranged from 0.08–1.12 g for SDW, 726.02–17,822.12 μg/g for SP, 0.18–9.15 mg for SPA, 0.45–2.10 for SDWR, 0.08–0.50 for SPR and 0.07–0.57 for SPAR, respectively. An analysis of variance (ANOVA) indicated that genotype and the genotype-by-environment interaction of three P efficiency-related traits and genotype of three relative values all existed significant difference (*P* < 0.001). The broad-sense heritability (*h*^*2*^) values of SDW, SP, SPA, SDWR, SPR and SPAR were 75.52–76.08%, 44.22–65.80%, 60.06–60.38%, 14.92%, 39.44% and 54.66%, respectively.

**Table 1:** Phenotypic values of three P efficiency related traits in two independent hydroponic experiments

Trait	Treatment	Exp.	Mean	CV%	Range	P^**a**^	G^**b**^	P × G^**c**^	***h***^***2***^/%^**d**^
SDW (g)	-P	E1	0.37 ± 0.13	34.55	0.08–0.76	***	***	***	75.52
	-P	E2	0.46 ± 0.16	35.23	0.12–0.90				
	+P	E1	0.40 ± 0.16	39.83	0.10–0.94				76.08
	+P	E2	0.49 ± 0.19	39.51	0.11–1.12				
SDWR	-P/+P	E1	0.98 ± 0.25	25.87	0.45–2.10	\	***	\	14.92
	-P/+P	E2	0.98 ± 0.25	25.78	0.46–1.70	\		\	
SP (μg/g)	-P	E1	2099.80 ± 632.34	30.11	846.27–4390.45	***	***	***	44.22
	-P	E2	1531.71 ± 487.81	31.85	726.02–3458.93				
	+P	E1	8786.83 ± 2188.60	24.91	4574.59–17,822.12				65.80
	+P	E2	7484.81 ± 1868.88	24.97	3683.65–15,166.40				
SPR	-P/+P	E1	0.24 ± 0.07	26.95	0.08–0.50	\	***	\	39.44
	-P/+P	E2	0.21 ± 0.07	32.32	0.09–0.42	\		\	
SPA (mg)	-P	E1	0.76 ± 0.31	40.23	0.19–1.80	***	***	***	60.38
	-P	E2	0.68 ± 0.30	43.74	0.18–2.17				
	+P	E1	3.41 ± 1.44	42.18	1.15–9.15				60.06
	+P	E2	3.54 ± 1.43	40.29	0.88–8.46				
SPAR	-P/+P	E1	0.24 ± 0.09	38.01	0.08–0.57	\	***	\	54.66
	-P/+P	E2	0.21 ± 0.09	42.58	0.07–0.47	\		\	

*SDW* shoot dry weight, *SDWR* the ratio of shoot dry weight under -P condition to +P condition, *SP* shoot P concentration, *SPR* the ratio of shoot P concentration under -P condition to +P condition, *SPA* shoot P accumulation, *SPAR* the ratio of shoot P accumulation under -P condition to +P condition. Exp.: Experiment; +P: One-half Hoagland’s solution supplied with 0.5 mM KH_2_PO_4_; −P: One-half Hoagland’s solution supplied with 0.005 mM KH_2_PO_4_. ^a^: Phosphorus level; ^b^: Genotype; ^c^: Phosphorus level × Genotype; ^d^: broad-sense heritability. ns: not significant; *, **and *** significant at 0.05, 0.01 and 0.001 probability levels, respectively

**Fig. 1:**
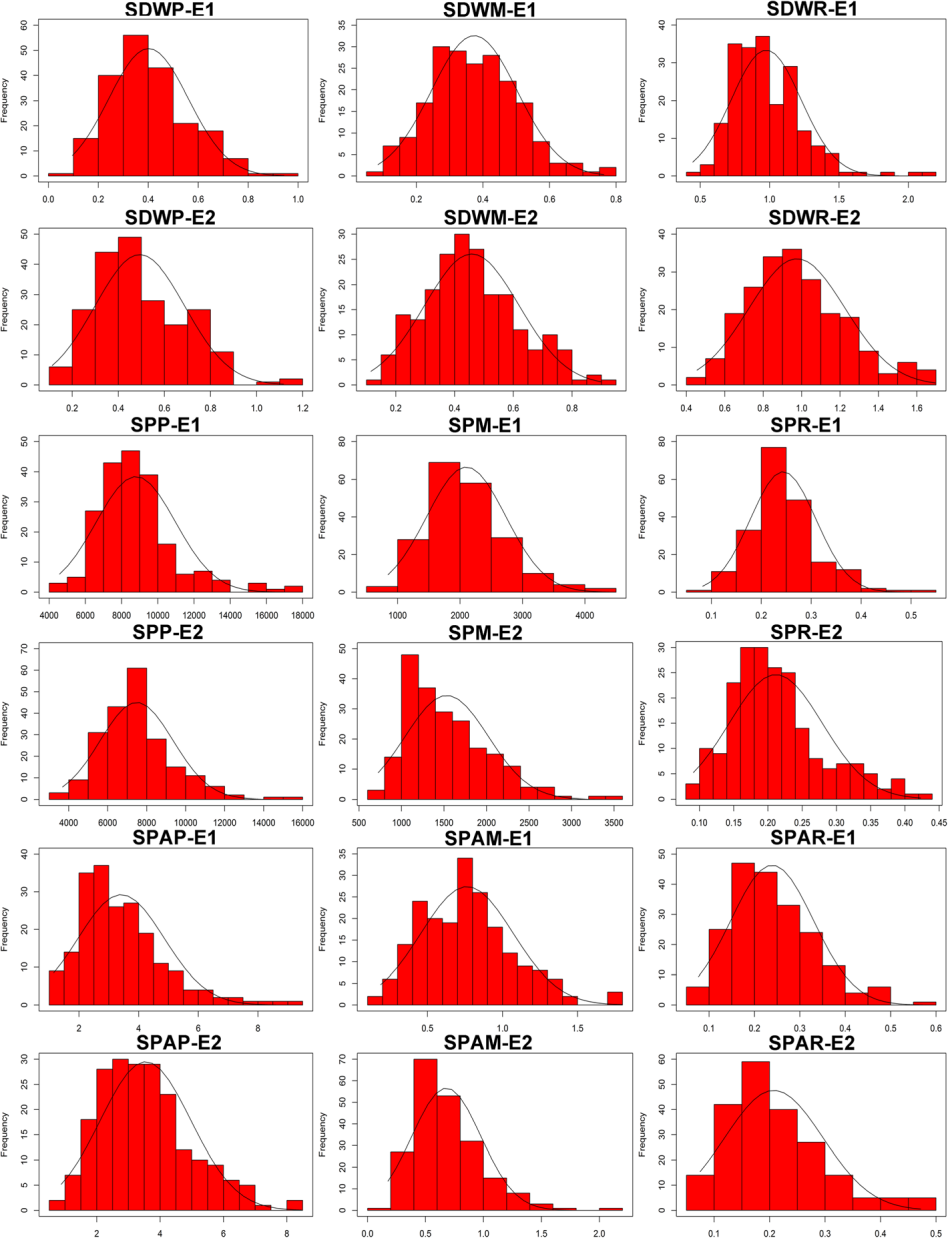
Frequency distribution of three P efficiency related traits in two independent hydroponic experiments. SDWP: shoot dry weight under +P condition, SDWM: shoot dry weight under -P condition, SDWR: the ratio of shoot dry weight under -P condition to +P condition; SPP: shoot P concentration under +P condition, SPM: shoot P concentration under -P condition, SPR: the ratio of shoot P concentration under -P condition to +P condition; SPAP: shoot P accumulation under +P condition, SPAM: shoot P accumulation under -P condition, SPAR: the ratio of shoot P accumulation under -P condition to +P condition. E1/E2: first/second independent hydroponic culture

The frequency distribution of three traits and their relative values in natural population was close to normal or near normal distribution, indicating that these three traits were quantitative traits (Fig. 1). The correlation coefficients of the mean value of three traits were analyzed in natural population (Additional file 2: Table S1). The three P efficiency-related traits were significantly correlated. SPA was strongly positively correlated with SDW and moderately positively correlated with SP. And SP was moderately negatively correlated with SDW. In summary, three P efficiency-related traits had large coefficients of variation. They were affected by accessions and environment and might be controlled by multiple genes.

### Genome-wide association analysis of P efficiency-related traits in soybean natural population

**Fig. 2:**
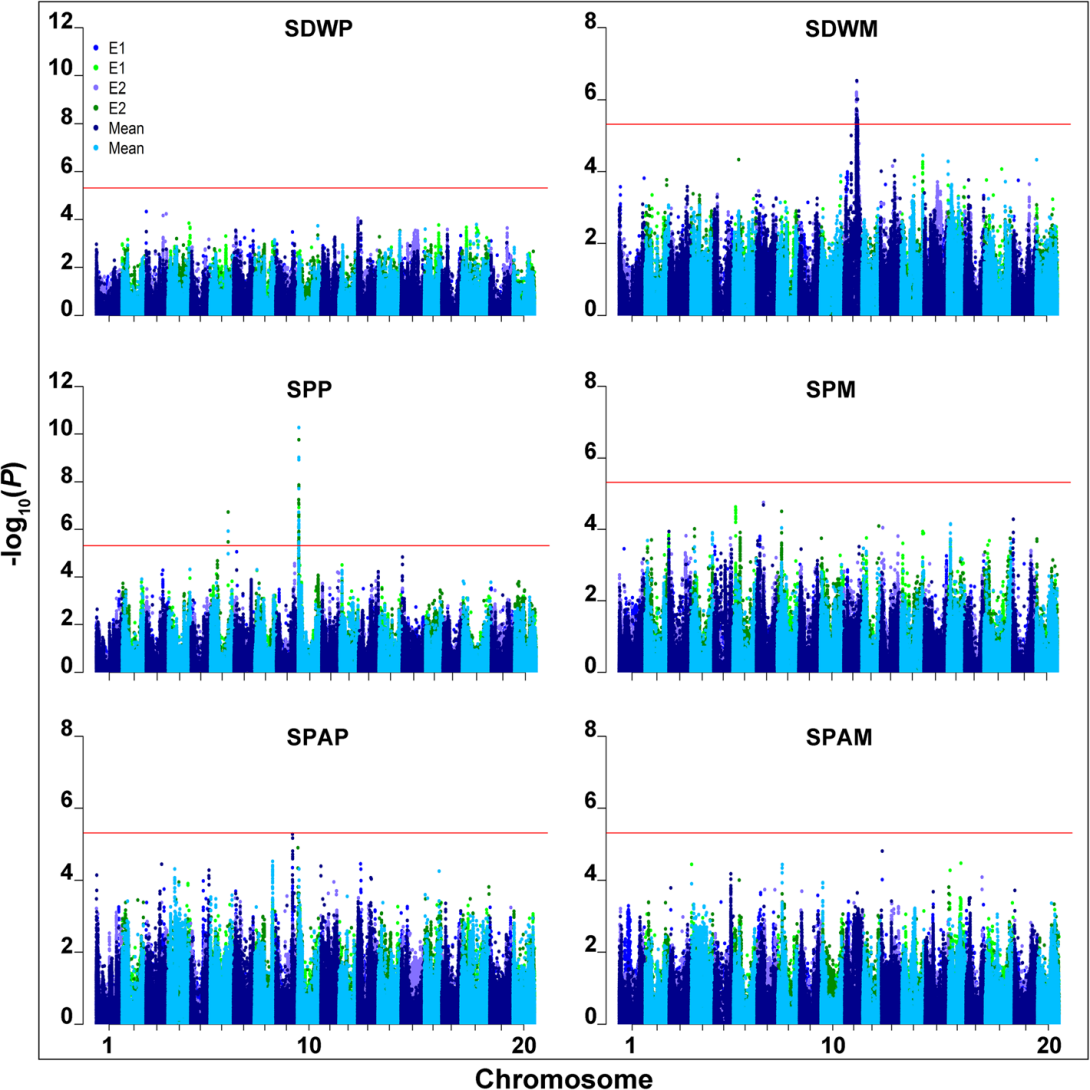
Manhattan plots of three P efficiency traits in two independent hydroponic experiments. SDWP: shoot dry weight under +P condition, SDWM: shoot dry weight under -P condition; SPP: shoot P concentration under +P condition, SPM: shoot P concentration under -P condition; SPAP: shoot P accumulation under +P condition, SPAM: shoot P accumulation under -P condition. The red line indicated the significance threshold (−log _10_ (*P*) =5.32). Manhattan plots of the same P efficiency traits in E1, E2 and their mean all in the same figure, blue and green plots represented manhattan plots of the same P efficiency traits in E1, lightslateblue and dark green plots represented manhattan plots of the same P efficiency traits in E2, and darkblue and deepskyblue plots represented manhattan plots of the same P efficiency traits in mean of E1 and E2

The values of three P efficiency-related traits of 211 soybean accessions under +P and -P conditions and the relative values of two traits were used for genome-wide association analysis (GWAS). The manhattan and quantile-quantile (QQ) plots for the GWAS results were shown in Fig. 2, Additional file 3: Figure S2 and Additional file 4: Figure S3. A total of 155 SNPs were significantly associated with SDW, SP and SPR (Table 2, Additional file 5: Table S2). Among them, 120 SNPs were detected only in one hydroponic experiment or the mean of E1 and E2 (Additional file 5: Table S2). Thirty-five SNPs were significantly associated with SDW or SP both in the mean of E1 and E2 of the same trait and at least one independent hydroponic experiment (Table 2), which were mainly located on chromosomes 6, 10 and 11 and clustered on chromosomes 10 and 11. There were 22 and 13 SNPs significantly associated with SDW and SP, respectively. The SNP AX-93733438 associated with SP under +P condition (SPP) on chromosome 6 fell into the genome region of three QTLs (*rdwnpC2–06*, *puenpC2–05* and *puenpC2–06,* with a same marker interval Satt489-Sat_251) [29]. Twelve SNPs associated with SPP on chromosome 10 fell into the genome region of two QTLs [*qCHL-O-1* (Satt487-Sat_108) and *qR/S-O-1* (Satt445-Satt487)] [34]. Among the significantly associated SNPs, four SNPs (AX-93636685, AX-93636692, AX-93932863, and AX-93932874) were significantly associated with SPP in E1, E2 and the mean of E1 and E2 (Table 2). Two SNPs, AX-93636685 and AX-93932874, were also significantly associated with SPR (Additional file 5: Table S2).

**Table 2:** Details of SNPs significantly associated with two P efficiency related traits in natural population

Trait	Marker	Chr.^**a**^	Position	***P*** value^**b**^	***R***^***2***^(%)^**c**^	Exp.^**d**^	Related QTLs in previous studies
SDWM	AX-93639768	11	17,540,742	2.98 × 10^− 06^-3.95 × 10^− 06^	6.92–6.00	E2, Mean^e^	
SDWM	AX-93793030	11	18,161,544	7.73 × 10^− 07^-4.00 × 10^− 06^	7.80–6.00	E2, Mean^e^	
SDWM	AX-93793042	11	18,125,598	7.50 × 10^−07^-2.57 × 10^− 06^	7.81–6.25	E2, Mean^e^	
SDWM	AX-93793048	11	18,114,663	6.91 × 10^− 07^-3.76 × 10^− 06^	7.87–6.03	E2, Mean^e^	
SDWM	AX-93793067	11	18,066,013	2.72 × 10^−06^-3.21 × 10^− 06^	6.98–6.12	E2, Mean^e^	
SDWM	AX-93793115	11	17,631,513	2.64 × 10^−06^-4.05 × 10^− 06^	7.00–5.99	E2, Mean^e^	
SDWM	AX-93793132	11	17,435,053	2.95 × 10^− 06^-4.19 × 10^− 06^	6.93–5.97	E2, Mean^e^	
SDWM	AX-93793133	11	17,434,154	4.65 × 10^−06^-4.57 × 10^− 06^	6.64–5.92	E2, Mean^e^	
SDWM	AX-93793134	11	17,413,322	2.45 × 10^−06^-2.99 × 10^−07^	7.05–7.49	E2, Mean^d^	
SDWM	AX-93793177	11	16,804,174	2.98 × 10^−06^-3.95 × 10^− 06^	6.92–6.00	E2, Mean^e^	
SDWM	AX-93793182	11	16,706,297	2.28 × 10^−06^-3.21 × 10^− 06^	7.09–6.12	E2, Mean^e^	
SDWM	AX-93793189	11	16,633,364	2.61 × 10^−06^-4.29 × 10^− 06^	7.01–5.96	E2, Mean^e^	
SDWM	AX-93793209	11	16,255,300	2.82 × 10^−06^-3.75 × 10^− 06^	6.96–6.03	E2, Mean^e^	
SDWM	AX-93793218	11	4,967,418	2.18 × 10^−06^-4.15 × 10^− 06^	7.12–5.98	E2, Mean^e^	
SDWM	AX-93793227	11	16,091,168	2.95 × 10^−06^-4.19 × 10^− 06^	6.93–5.97	E2, Mean^e^	
SDWM	AX-94089576	11	18,592,188	1.02 × 10^−06^-4.30 × 10^− 06^	7.61–5.95	E2, Mean^e^	
SDWM	AX-94089731	11	18,163,010	6.31 × 10^−07^-2.57 × 10^−06^	7.93–6.20	E2, Mean^e^	
SDWM	AX-94089820	11	17,510,382	9.65 × 10^−07^-9.73 × 10^− 07^	7.65–6.81	E2, Mean^e^	
SDWM	AX-94089832	11	17,366,064	3.19 × 10^−07^-1.97 × 10^−06^	8.37–6.40	E2, Mean^e^	
SDWM	AX-94089845	11	17,134,392	2.95 × 10^−06^-4.19 × 10^− 06^	6.93–5.97	E2, Mean^e^	
SDWM	AX-94089894	11	16,493,472	2.34 × 10^−06^-4.55 × 10^− 06^	7.08–5.92	E2, Mean^e^	
SDWM	AX-94089934	11	15,954,738	2.98 × 10^−06^-3.95 × 10^− 06^	6.92–6.00	E2, Mean^e^	
SPP	AX-93733438	6	38,730,523	1.84 × 10^−07^-1.18 × 10^−06^	12.33–10.28	E2, Mean^e^	*rdwnpC2–06*, *puenpC2–05*, *puenpC2–06* (Satt489-Sat_251)
SPP	AX-93636685	10	1,592,679	2.95 × 10^−07^-1.18 × 10^−09^	12.64–16.65	E1, E2, Mean^e^	*qCHL-O-1* (Satt487-Sat_108), *qR/S-O-1* (Satt445-Satt487)
SPP	AX-93636692	10	1,606,922	2.07 × 10^−06^-1.92 × 10^−08^	10.73–14.01	E1, E2, Mean^e^	
SPP	AX-93932863	10	1,591,041	2.48 × 10^−07^-9.63 × 10^−10^	12.81–16.84	E1, E2, Mean^e^	
SPP	AX-93932874	10	1,627,680	1.21 × 10^−07^-5.36 × 10^−11^	13.53–19.65	E1, E2, Mean^e^	
SPP	AX-93636690	10	1,605,716	1.23 × 10^−06^-5.96 × 10^−07^	10.57–10.88	E2, Mean^e^	
SPP	AX-93774132	10	1,496,425	5.57 × 10^−08^-4.29 × 10^−07^	13.45–11.18	E2, Mean^e^	
SPP	AX-93774138	10	1,507,397	5.57 × 10^−08^-4.29 × 10^−07^	13.45–11.18	E2, Mean^e^	
SPP	AX-93774183	10	1,604,160	3.17 × 10^−06^-3.41 × 10^−06^	9.71–9.34	E2, Mean^e^	
SPP	AX-93774188	10	1,616,592	2.36 × 10^−07^-3.96 × 10^− 07^	12.09–11.25	E2, Mean^e^	
SPP	AX-93932870	10	1,606,515	3.38 × 10^−06^-4.57 × 10^− 06^	9.65–9.09	E2, Mean^e^	
SPP	AX-94070704	10	1,510,255	7.67 × 10^−08^-1.87 × 10^−07^	13.15–11.92	E2, Mean^e^	
SPP	AX-94070763	10	1,625,676	6.27 × 10^−07^-1.66 × 10^−06^	11.19–9.97	E2, Mean^e^	

*SDWP* shoot dry weight under +P condition, *SDWM* shoot dry weight under -P condition, *SPP* shoot P concentration under +P condition, *SPM* shoot P concentration under -P condition, *SPAP* shoot P accumulation under +P condition, *SPAM* shoot P accumulation under -P condition. Position: soybean genome assembly *Glycine max Wm82.a2.v1*. E1/E2: first/second independent hydroponic culture. ^a^: chromosome; ^b^: significant at *P* ≤ 4.82 × 10^−6^; ^c^: percentage of phenotypic variation explained by the SNP; ^d^: experiment; ^e^: Mean, average of E1 and E2

### Identification of favorable haplotypes related to shoot P concentration

**Fig. 3:**
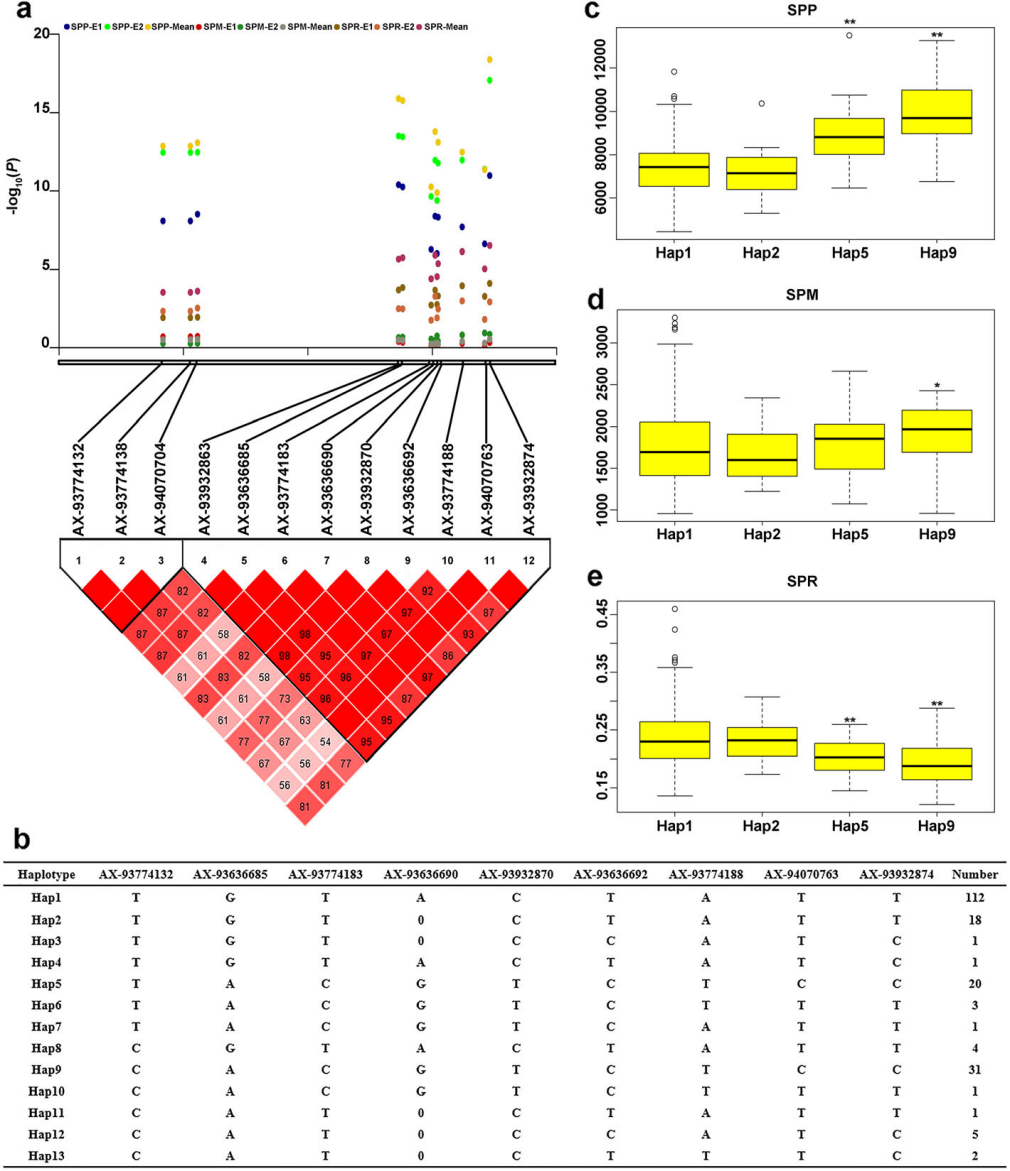
Haplotype analysis of twelve SNPs significantly associated with SP and SP value of different haplotypes. **a** The physical locations of 12 SNPs and the pairwise LD analysis between these SNPs. The color squares was used to indicate the LD indexed. Red squares without numbers indicated complete LD (D’ = 1, *P* < 0.01). D’ values were shown in the squares for values < 1.0. **b** Haplotypes of 200 natural population using the 12 SNPs; 0 indicates a base deletion. **c** SP among four haplotypes Hap1, Hap2, Hap5 and Hap9 under +P condition. **d** SP among four haplotypes Hap1, Hap2, Hap5 and Hap9 under -P condition. **e** The relative value of SP among four haplotypes Hap1, Hap2, Hap5 and Hap9 under two P conditions. Statistical significance was detected by a two-tailed t-test. * and ** significant at 0.05 and 0.01 probability levels, respectively

Four SNPs on chromosome 10 were significantly associated with SPP in two hydroponic experiments and mean of E1 and E2, and two of them were also significantly associated with SPR. There were also eight SNPs on chromosome 10 significantly associated with SPP in E2 and mean of E1 and E2. These twelve SNPs explained 9.09–19.65% of the phenotypic variation (Table 2, Additional file 5: Table S2). Therefore, these 12 SNPs were used for the analysis of haplotypes of shoot P concentration in 200 accessions. Twelve SNPs exhibited strong linkage disequilibrium (LD) and formed two LD blocks (Fig. 3a). Among them, AX-93774132, AX-93774138 and AX-94070704 showed complete LD. AX-93932863 and AX-93636685 also showed complete LD. Therefore, these 12 SNPs were subdivided into nine SNPs and were classified into 13 haplotypes (Fig. 3b). Haplotype 1 (Hap1, *n* = 112) formed the largest group, Hap9 (*n* = 31) was the second largest group, Hap5 (*n* = 20) was the third largest group, Hap2 (*n* = 18) was the fourth largest group and the other nine haplotype classes constituted minor groups. The soybeans with Hap5 or Hap9 had significantly higher SP under +P condition, compared with that of Hap1 or Hap2; and the SP of soybean with Hap9 was significantly higher than that of Hap5 (Fig. 3c). Under -P condition, there was no significant difference, except that the SP of soybean with Hap9 was significantly higher than that of Hap2 (Fig. 3d). The relative value of SP in soybean with Hap5 or Hap9 was significantly lower than that of Hap1 or Hap2, owing to higher SP under +P condition (Fig. 3e). Therefore, soybean with Hap9 had higher SP under two P conditions.

### Identification of candidate gene for P efficiency

According to the calculation of the linkage disequilibrium distance [33], genes within 130 Kb upstream and downstream of SNPs were analyzed. Four SNPs (AX-93636685, AX-93636692, AX-93932863, and AX-93932874) were significantly associated with SPP, and two of them (AX-93636685 and AX-93932874) were also significantly associated with SPR. Among them, two SNPs (AX-93932863 and AX-93636692) fell within intergenic region. It was noteworthy that the remaining two SNPs (AX-93636685 and AX-93932874) fell within two genes respectively. The SNP (AX-93636685) was detected in intron of *Glyma.10 g018200*, and the other SNP (AX-93932874) was detected in 5′-untranslated region of *Glyma.10 g018800*. Therefore, *Glyma.10 g018200* and *Glyma.10 g018800* were selected for further functional analysis.

**Fig. 4:**
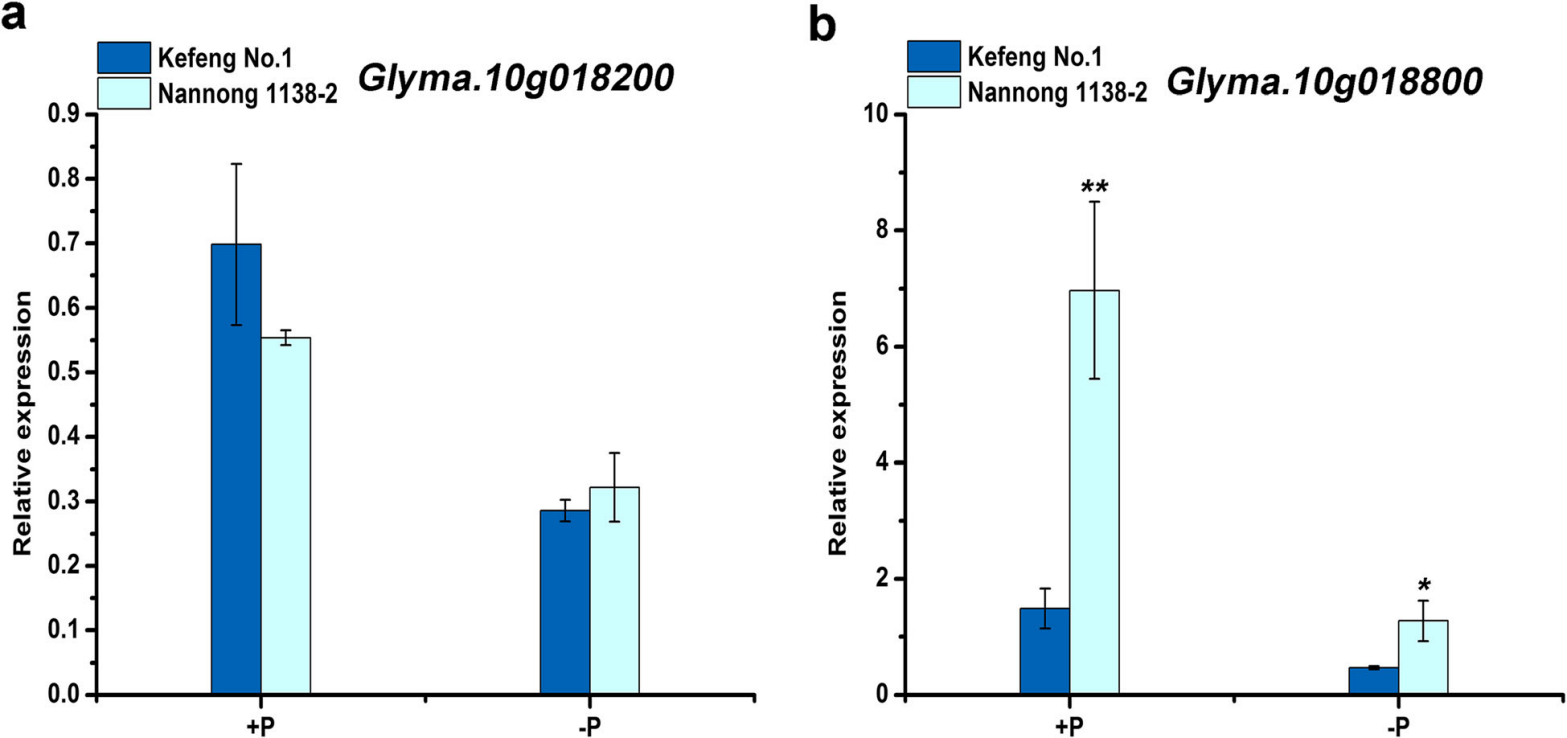
Expression of *Glyma.10 g018200* and *Glyma.10 g018800* in Kefeng No. 1 and Nannong 1138–2. **a** qRT-PCR analysis of *Glyma.10 g018200* transcription levels in Kefeng No. 1 and Nannong 1138–2. **b** qRT-PCR analysis of *Glyma.10 g018800* transcription levels in Kefeng No. 1 and Nannong 1138–2. The seeds were germinated for 4 days in vermiculite, and uniform seedlings were selected and cultured for 3 days in one-half Hoagland's nutrient solution containing 0.5 mM KH_2_PO_4_. Finally, they were transferred to +P (0.5 mM KH_2_PO_4_) and -P (0.005 mM KH_2_PO_4_) nutrient solution for 15 days and then sampled. Tubulin was used as an internal control. Data were the mean values of biological replicates mean ± standard deviation (SD) (*n* = 3). Statistical significance was detected by a two-tailed t-test. * and ** significant at 0.05 and 0.01 probability levels, respectively

To determine whether the transcriptional level of these two genes (*Glyma.10 g018200* and *Glyma.10 g018800*) were affected by low P stress, the relative expression levels were examined in soybean accessions Kefeng No. 1 and Nannong 1138–2 (Fig. 4). According to previous reports, Kefeng No. 1 showed higher P efficiency than that of Nannong 1138–2 [28]. The relative expression of these two genes decreased significantly under -P condition, compared to +P condition. However, the relative expression of *Glyma.10 g018200* was not significantly different between these two soybean cultivars, whether under +P or -P conditions (Fig. 4a). In contrast, the relative expression of *Glyma.10 g018800* was significantly different in Nannong 1138–2 and Kefeng No.1 under +P and -P conditions (Fig. 4b). The relative expression of *Glyma.10 g018800* in Nannong 1138–2 was 4.7 times that of Kefeng No.1 under +P condition and 2.7 times that of Kefeng No.1 under -P condition.

Therefore, the relative expression of *Glyma.10 g018800* was significantly different in Nannong 1138–2 and Kefeng No.1, indicating that the relative expression of this gene might be highly correlated with soybean P efficiency. Taken together, *Glyma.10 g018800* was chosen as a candidate gene for further research in this study.

### *Glyma.10 g018800* belonged to SPX-RING family and was named as *GmSPX-RING1*

**Fig. 5:**
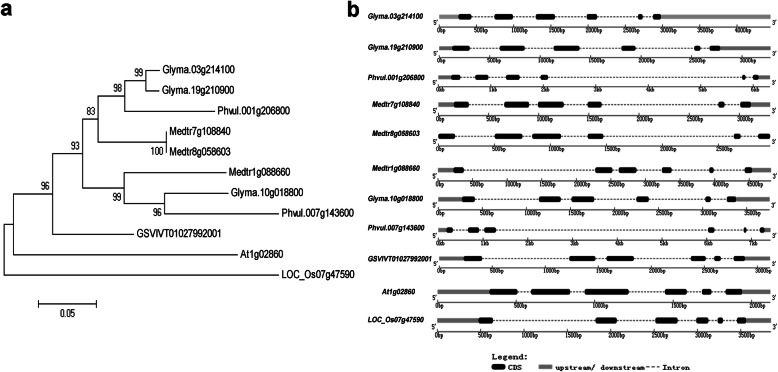
Phylogenetic tree and Exon/intron structure in six species. **a** Phylogenetic tree analysis. Glyma.03 g214100, Glyma.10 g018800 and Glyma.19 g210900 were from soybean [*Glycine max* (L.) Merr.]; LOC_Os07g47590 were from rice (*Oryza sativa*); At1g02860 were from *Arabidopsis* (*Arabidopsis thaliana*); Phvul.001 g206800 and Phvul.007 g143600 were from common bean (*Phaseolus vulgaris*); Medtr7g108840, Medtr8g058603 and Medtr1g088660 were from alfalfa (*Medicago truncatula*); GSVIVT01027992001 was from grape (*Vitis vinifera*). **b** Gene structure analysis. Black boxes represented exons and dashed lines represented introns

The full-length open reading frame (ORF) of *Glyma.10 g018800* was 948 bp and encoded 315 amino acids with a calculated mass of 36.12 KDa and a pI of 8.76. To further understand the candidate gene, full-length protein sequence of *Glyma.10 g018800* was blasted on Phytozome v12.1 (https://phytozome.jgi.doe.gov/pz/portal.html) in *Arabidopsis* (*Arabidopsis thaliana*), common bean (*Phaseolus vulgaris*), alfalfa *(Medicago truncatula),* grape (*Vitis vinifera*) and rice (*Oryza sativa*) to find homologous proteins with E values less than 10^− 100^. Phylogenetic tree was drawn after multiple sequence alignments of homologous proteins in six species (Fig. 5a). And the structure analysis showed that all the 11 genes included 6 exons and 5 introns (Fig. 5b). The results showed that Glyma.10 g018800 was highly conserved in six plants.

Amino acid sequence alignment showed that the similarity between Glyma.10 g018800 and At1g02860 was 64.78%, and the similarity with LOC_Os07g47590 was 62.85% (Additional file 6: Figure S4a). After searching 11 genes in NCBI (https://www.ncbi.nlm.nih.gov/), it was found that *At1g02860* named *AtNLA* in *Arabidopsis* and *LOC_Os07g47590* named *OsNLA1* in rice had been studied. *AtNLA* and *OsNLA1* were involved in maintenance of phosphate homeostasis [19, 22, 35]. Like *AtNLA* and *OsNLA1*, *Glyma.10 g018800* also contained an N-terminal SPX domain and a C-terminal RING domain (Additional file 6: Figure S4b). Therefore, *Glyma.10 g018800*, which contained SPX and RING domains, was named as *GmSPX-RING1*. And *GmSPX-RING1* might be responsible for regulating the P efficiency in different cultivars.

To further understand the expression pattern of *GmSPX-RING1*, the relative expression was detected in different tissues of soybean (Additional file 7: Figure S5). *GmSPX-RING1* was mainly expressed in roots, and the relative expression of *GmSPX-RING1* was low in flowers, pods, leaves, stems and seeds.

### GmSPX-RING1 was localized in cell membrane

**Fig. 6:**
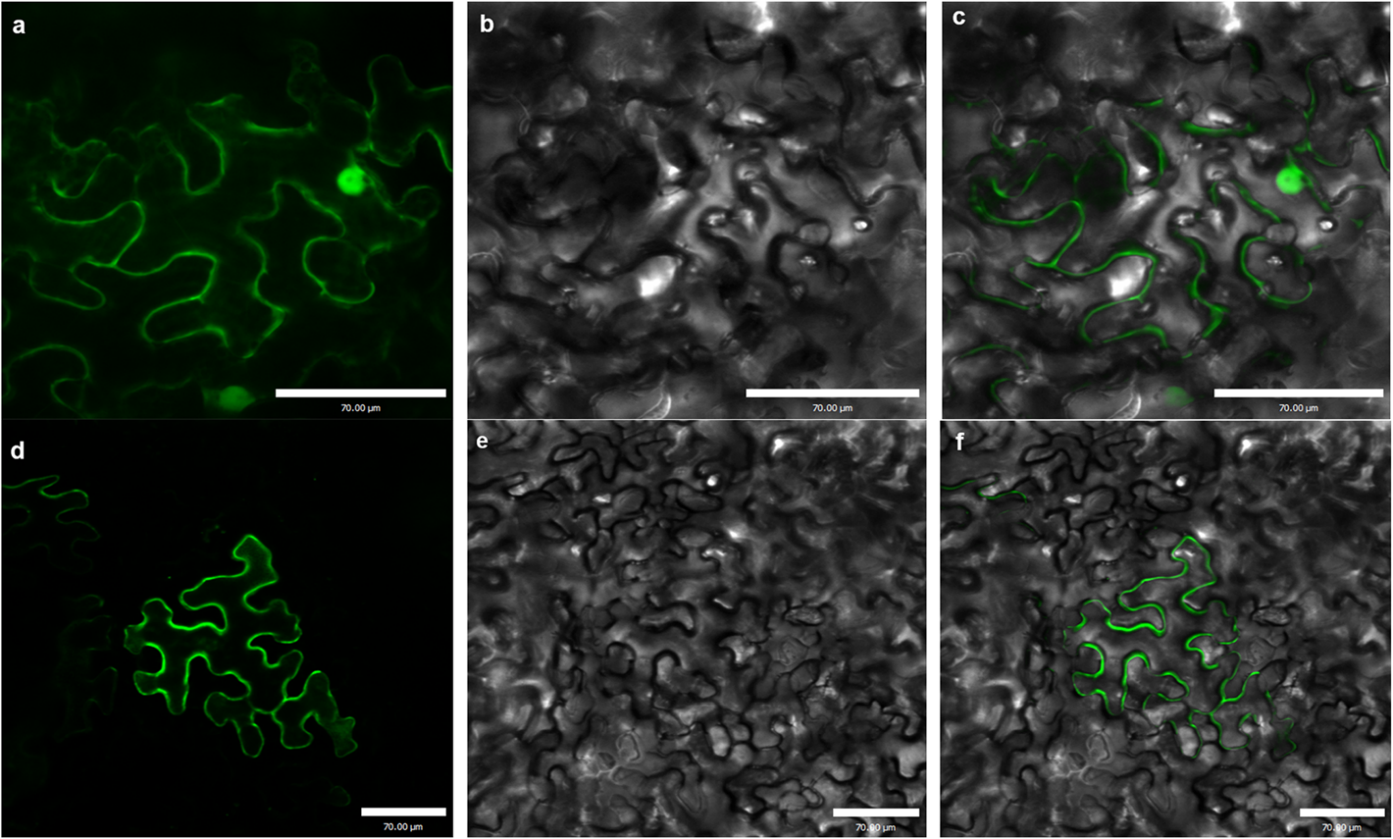
Subcellular localization of GmSPX-RING1-GFP Protein. Individual panels showed GFP alone **a** or GmSPX-RING1-GFP **d** in tobacco leaf cells, corresponding bright-field images **b** and **e**, and merged images **c** and **f**, respectively. GFP and GmSPX-RING1-GFP fusion was driven by the control of the CaMV 35S promoter. Bars = 70.00 μm

To determine the subcellular location of the GmSPX-RING1 protein, the CDS sequence of GmSPX-RING1 on a vector controlled by the CaMV 35S promoter was constructed. The recombinant constructs of the GmSPX-RING1-GFP fusion and GFP alone were introduced into tobacco leaves through *Agrobacterium* inoculation. The GmSPX-RING1-GFP fusion protein was observed primarily in the tobacco cell membrane (Fig. 6d, e, f), while the GFP protein was observed throughout the cell (Fig. 6a, b, c). GmSPX-RING1 was primarily localized in cell membrane in tobacco which was similar to AtNLA [22] and OsNLA1 [19].

### *GmSPX-RING1* affected P concentration in soybean hairy roots

**Fig. 7:**
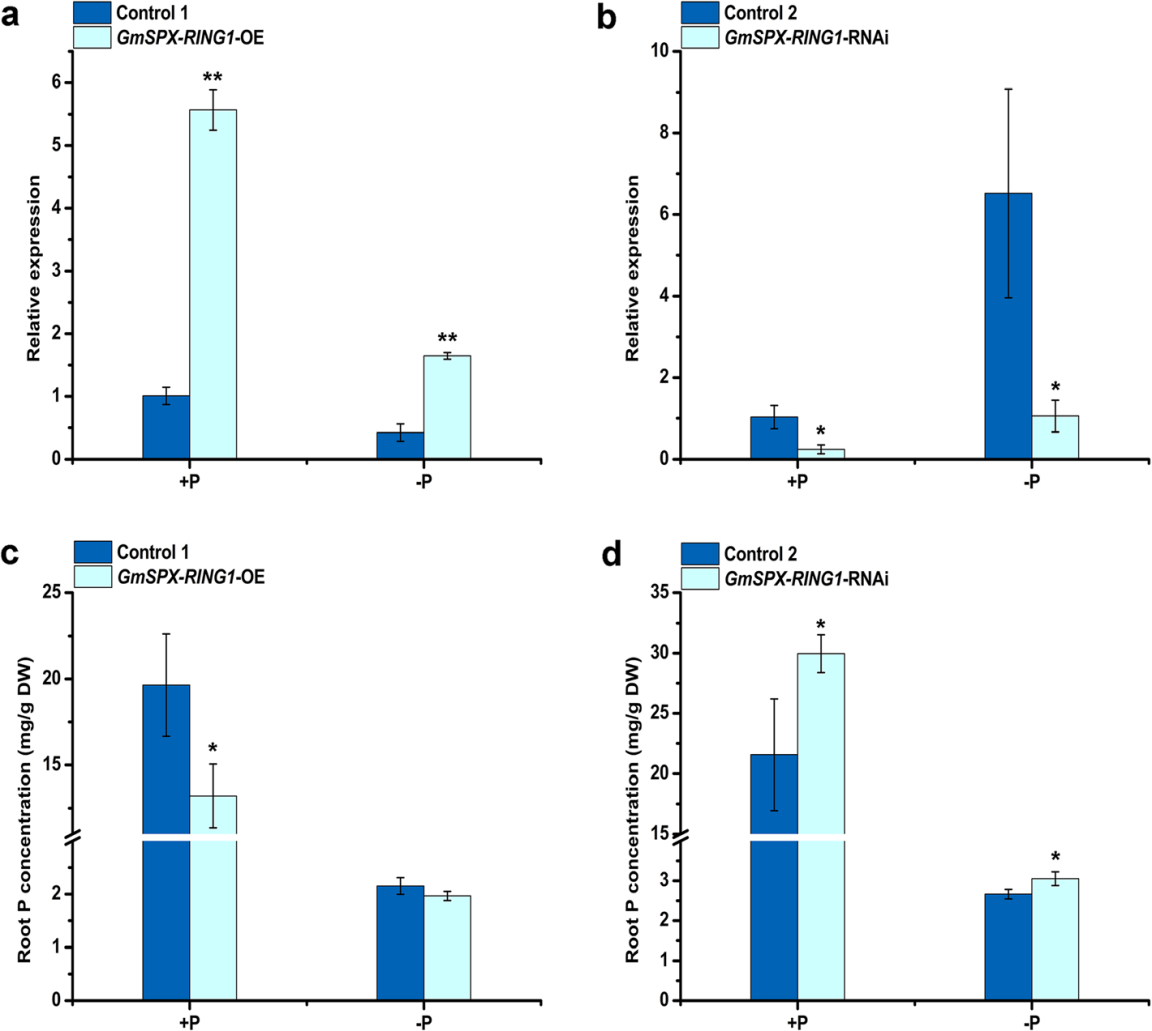
The relative expression of *GmSPX-RING1* and P concentration in *GmSPX-RING1*-OE and *GmSPX-RING1-*RNAi transgenic hairy roots. **a** Relative expression of *GmSPX-RING1* in *GmSPX-RING1*-OE and Control 1 transgenic hairy roots. **b** Relative expression of *GmSPX-RING1* in *GmSPX-RING1-*RNAi and Control 2 transgenic hairy roots. **c** P concentration in *GmSPX-RING1*-OE and Control 1 transgenic hairy roots. **d** P concentration in *GmSPX-RING1-*RNAi and Control 2 transgenic hairy roots. *GmSPX-RING1*-OE: soybean transgenic hairy roots with *GmSPX-RING1* overexpression vector, Control 1: soybean transgenic hairy roots with overexpression empty vector; *GmSPX-RING1*-RNAi: soybean transgenic hairy roots with RNA interference of *GmSPX-RING1* vector, Control 2: soybean transgenic hairy roots with RNA interference empty vector. *GmSPX-RING1*-OE and *GmSPX-RING1-*RNAi hairy roots and their controls grown under +P or -P condition for 15d. Data were the mean values of biological replicates mean ± standard deviation (SD) (*n* = 3). Statistical significance was detected by a two-tailed t-test. * and ** significant at 0.05 and 0.01 probability levels, respectively

To further understand the function of *GmSPX-RING1* in soybean, overexpression (*GmSPX-RING1*-OE) and RNA interference of *GmSPX-RING1* (*GmSPX-RING1*-RNAi) in soybean were achieved by *Agrobacterium rhizogenes*-mediated hairy root transformation systems. Transgenic hairy roots were screened by green fluorescence and hairy roots containing green fluorescence were used for RNA extraction and subsequent determination of P concentration (Additional file 8: Figure S6). The relative expression of *GmSPX-RING1* in *GmSPX-RING1*-OE hairy root was 5.53 times that of Control 1 under +P condition and 3.89 times that of Control 1 under -P condition (Fig. 7a). In *GmSPX-RING1*-RNAi hairy root, the relative expression of *GmSPX-RING1* was 0.24 times that of Control 2 under +P condition and 0.16 times that of Control 2 under -P condition (Fig. 7b).

To investigate the effect of *GmSPX-RING1* in soybean hairy roots, fresh weight of hairy roots and the root P concentration of *GmSPX-RING1*-OE, *GmSPX-RING1*-RNAi and their respective controls were examined. Under both +P and -P conditions, there was no significant difference in fresh weight of hairy roots between *GmSPX-RING1*-OE and Control 1 (Additional file 9: Figure S7a). Compared to Control 2, fresh weight of hairy roots in *GmSPX-RING1*-RNAi significant reduced under +P conditions but was no significant difference under -P conditions (Additional file 9: Figure S7b). Then, the P concentration in *GmSPX-RING1*-OE and *GmSPX-RING1*-RNAi transgenic hairy roots was measured. In soybean *GmSPX-RING1*-OE lines, the P concentration in hairy roots significantly reduced by 32.75% compared to that of the Control 1 under +P condition (Fig. 7c). And, in *GmSPX-RING1*-RNAi lines, P concentration in hairy roots increased by 38.90% significantly compared to that of Control 2 under +P condition (Fig. 7d). Under -P condition, the P concentration in *GmSPX-RING1*-RNAi hairy roots increased by only 14.51% compared to that of Control 2 and there was no P concentration significant difference between *GmSPX-RING1*-OE and Control 1 hairy roots. These results indicated that *GmSPX-RING1* negatively regulated P concentration in soybean transgenic hairy roots.

## Discussion

Soybean requires large amounts of P from vegetative growth to reproductive growth [25]. However, the phosphate of the soil is difficult to be absorbed and utilized by plants, resulting in genetically P deficient in plants. Therefore, increasing the PAE by increasing the absorption of poorly soluble phosphate in the soil and PUE by distribution and remobilization of acquired phosphate is essential for plant breeding.

### Loci significantly associated with phosphorus efficiency and candidate gene of interest were identified

Genetic mapping through linkage or association analysis requires high genetic diversity at the phenotype and DNA levels [36]. GWAS is an effective method to identify key genes in crops, such as corn [37] and soybeans [38]. In this study, three shoot P efficiency-related traits and relative values of two traits combined with NJAU 355 K SoySNP array were used for GWAS. The NJAU 355 K SoySNP array was also used to identify critical transcription factor *GmMYB29* related to isoflavone content [39] and *GmCDF1* related to salt tolerance [40]. There were a total of 155 SNPs significantly associated with P efficiency-related traits (Table 2, Additional file 5: Table S2). Among them, 112 SNPs significantly associated with SDW on chromosome 11 ranged from 15,446,698 to 18,657,407 (about 3.2 Mb), which were not reported in previous studies, representing novel loci related to P efficiency. Forty SNPs significantly associated with SP on chromosome 10 ranged from 1,485,231 to 1,627,680 (about 142 Kb). These SNPs fell into the genome region of *qCHL-O-1* (Satt487-Sat_108) and *qR/S-O-1* (Satt445-Satt487) [34]. Two SNPs on chromosome 6, AX-93733438 and AX-94029729, fell into the genome region of three QTLs *rdwnpC2–06*, *puenpC2–05* and *puenpC2–06* (with a same marker interval Satt489-Sat_251) [29]. These novel and co-location SNPs identified in this study were helpful for further understanding the genetic basis of soybean P efficiency.

Four SNPs (AX-93932863, AX-93636685, AX-93636692, and AX-93932874) on chromosome 10 were significantly associated with SPP in two hydroponic experiments, and two of them (AX-93636685 and AX-93932874) were significantly associated with SPR through GWAS analysis (Table 2, Additional file 5: Table S2). Interestingly, the SNP (AX-93932874) was identified to fall in 5′-untranslated region of *GmSPX-RING1.* The 5’UTR sequence of *GmSPX-RING1* was predicted by PLACE and found that the SNP was located in TGAC-motif, which was core motif of *WRKY* transcription factor family binding site W-box. *WRKY* transcription factors played important roles in biotic and abiotic stresses, and *WRKY6* [41], *WRKY45* [42], and *WRKY75* [43] had been found to be associated with P stress in *Arabidopsis*. In addition, the 5’UTR mainly affected the regulation of genes by affecting mRNA stability, folding and interaction with ribosomal mechanisms through the post-transcriptional stages [44]. So, the SNP in 5’UTR may affect the regulation of *GmSPX-RING1* transcription or post-transcriptional by *WRKY* transcription factors, but the regulatory mechanism needs further study.

### *GmSPX-RING1* negatively regulated phosphorus content in soybean transgenic hairy roots

Most of genes containing SPX domain (SYG1/PHO81/XPR1) in plants are associated with phosphate homeostasis, such as *SPX* and *PHO1*. There are four families containing the SPX domain, named after the added domains: SPX, SPX-MFS, SPX-EXS and SPX-RING. The only characterized member of the SPX-RING family in *Arabidopsis* and rice was *Nitrogen Limitation Adaptation* (*NLA*). *NLA* containing both SPX and RING domains was involved in P uptake and P content homeostasis [18]. Conserved domain prediction of the GmSPX-RING1 amino acid sequence revealed that it contained SPX domain and RING domain (Additional file 6: Figure S4). Gene structure and phylogenetic tree analysis results showed that *GmSPX-RING1* was conserved in six species (Fig. 5).

To further understand whether *GmSPX-RING1* affected phosphate acquisition and transport in soybean, soybean hairy root transformation was performed. Overexpression of *GmSPX-RING1* (*GmSPX-RING1*-OE) reduced P concentration significantly in soybean hairy roots compared with that of Control 1 under +P condition. And RNA interference of *GmSPX-RING1* (*GmSPX-RING1*-RNAi) increased P concentration significantly in soybean hairy roots, both under +P and -P conditions compared with that of Control 2 (Fig. 7c, d). The P concentration of *GmSPX-RING1*-OE hairy roots did not decrease significantly compared with that of Control 1 under -P condition, which might due to low P concentration in nutrient solution. Although the P concentration of *GmSPX-RING1*-RNAi hairy root increased significantly under -P condition, P concentration in *GmSPX-RING1*-RNAi hairy roots was 1.15 times that of Control 2, with only a slight increase. Similar results were also found in rice [19]. P toxicity was observed in mature leaf tips of *osnla1* mutant, and P concentration of *osnla1* was more than four times that of WT under +P condition. P toxicity did not occur under -P condition, and P concentration of only part of leaves increased. However, P concentration of overexpressing lines showed a significant decrease in some leaves only under +P condition, and there was no significant difference compared with WT under -P condition [19]. These results indicate that the relative expression of *GmSPX-RING1* is negatively correlated with the P concentration in soybean hairy roots. *GmSPX-RING1* may negatively regulate P uptake as well as *AtNLA* and *OsNLA1* [19, 22, 35].

## Conclusions

In this study, 155 SNPs were significantly associated with P efficiency traits through GWAS. In addition, 13 haplotypes (Hap1-Hap13) related to shoot P concentration were identified and Hap9 was considered as the optimal haplotype. Four SNPs (AX-93636685, AX-93636692, AX-93932863, and AX-93932874) located on chromosome 10 were identified to be significantly associated with shoot P concentration under +P condition in two hydroponic experiments. One of these four SNPs fell in the 5′-untranslated region of *Glyma10g018800*. *Glyma10g018800* contained SPX and RING domains and was named as *GmSPX-RING1*. Through induced expression, candidate gene *GmSPX-RING1* was confirmed. Overexpression and RNA interference of *GmSPX-RING1* indicated that *GmSPX-RING1* negatively regulated P concentration in transgenic hairy roots. The SNPs could be potentially used in identification of P efficiency genes and high P efficiency breeding in soybean.

## Methods

### Plant materials and hydroponic culture

A natural population containing 211 soybean [*Glycine max* (L.) Merr.] accessions with diverse geographic origins were collected (Additional file 10: Table S3) [33]. Among them, 191 accessions originated from China. Ten accessions originated from other countries, i.e., one from Brazil, two from Japan and seven from America. The origination of the remaining ten accessions were unknown. This population included 180 landraces and 23 improved soybeans. The remaining eight accessions were ecologically unknown. Seeds of this population were supplied by National Center for Soybean Improvement (Nanjing Agricultural University, Nanjing, China).

The hydroponic culture experiments were conducted in a greenhouse at Nanjing Agricultural University (Nanjing, China). Seeds were sterilized in Cl_2_ for 5 h and then germinated in sterilized vermiculites. After 3–4 days of germination, six replicates of each accession were selected and transferred to one-half Hoagland’s solution. Three replicates of one accession were grown in one-half Hoagland’s solution supplemented with 0.5 mM KH_2_PO_4_ (+P, normal P condition), and the other three replicates were grown in one-half Hoagland’s solution supplemented with 0.005 mM KH_2_PO_4_ (−P, P deficiency condition). Under -P condition, KH_2_PO_4_ was replaced by equal concentration of KCl. One-half Hoagland’s solution was composed of macroelements [2.5 mM Ca(NO_3_)_2,_ 2.5 mM KNO_3_, 1.0 mM MgSO_4_, 0.5 mM KH_2_PO_4_ /0.5 mM KCl] and microelements (10 μM EDTA Na_2_, 10 μM FeSO_4,_ 23 μM H_3_BO_3_, 4.5 μM MnCl_2_, 0.15 μM CuSO_4_, 0.4 μM ZnSO_4_, 0.05 μM Na_2_MoO_4_). The pH of the nutrient solution was adjusted to 5.8–6.0. They were grown in a green house with a 12 h/12 h photoperiod and a temperature of 30–32 °C/22 °C (day/night). The 20 L hydroponic tanks (57.8 × 38.6 × 15.5, length×width×height, cm) were used and for each tank a supporting plate containing 59 holes was used to plant soybean seedlings. A randomized blocks design was employed. Nutrient solution was changed every three days. The shoots (cotyledon node to the top growth point) were sampled at 15d after transfer. Two independent hydroponic experiments were conducted: The first experiment (E1) was from April to June, 2015, and the second experiment (E2) was from May to July, 2016.

### Measurement of phenotypic traits

The samples (shoots of 211 soybean accessions) were oven-dried at 105 °C for 1 h and then at 65 °C to constant weight to weigh shoot dry weight (SDW) [30]. Shoot dry weight (SDW) under +P condition was abbreviated as SDWP; shoot dry weight (SDW) under -P condition was abbreviated as SDWM; the ratio of SDW under -P condition to +P condition was abbreviated as SDWR. The soybean transgenic hairy roots were also oven-dried to constant weight. The dry samples were ground into powder and weighed about 0.1 g for digestion. Then 2 mL of pure HNO_3_ was added to the samples (about 0.1 g) and the samples were digested with a microwave digestion system (Milestone Ethos) for 25 min. After cooling, the digested sample was transferred to a volumetric flask to a volume of 25 mL. And 10 mL from the 25 mL sample was transferred to the tube and stored at − 20 °C for further measurement. The shoot P concentration (SP) of the samples was measured using Perkin Elmer Optima 8000 ICP-OES system. Shoot P concentration (SP) under +P condition was abbreviated as SPP; shoot P concentration (SP) under -P condition was abbreviated as SPM; the ratio of SP under -P condition to +P condition was abbreviated as SPR. The total P content in shoot was named as shoot P accumulation (SPA) = shoot dry weight (SDW) × shoot P concentration (SP); shoot P accumulation (SPA) under +P condition was abbreviated as SPAP; shoot P accumulation (SPA) under -P condition was abbreviated as SPAM; the ratio of SPA under -P condition to +P condition was abbreviated as SPAR.

### Statistical analysis

Mean, coefficient of variation (CV), analysis of variance (ANOVA) and correlation analyses were conducted using SAS 9.2 (SAS Institute Inc., USA). The broad-sense heritability estimates (*h*^*2*^) was defined by Shi et al. [45] and conducted using SAS 9.2 (SAS Institute Inc., USA). Histogram was conducted using Origin Pro 8.5. Manhattan plots, QQ plots, frequency distribution histogram and box plots were all conducted with R.

### GWAS for P efficiency-related traits at seedling stage

The mean values of three shoot P efficiency traits (SDW, SP and SPA) after hydroponics for 15 days under two P conditions (0.5 mM and 0.005 mM KH_2_PO_4_) of E1, E2 and the relative phenotypic values of these traits (SPR and SPAR) were used for GWAS. GWAS was performed using phenotypic values and 207,607 SNPs with a minor allele frequency MAF > 0.05, which was the result of genotyping of NJAU 355 K SoySNP array in these 211 accessions [33]. GWAS was conducted with the GAPIT package [46] using a compressed mixed linear model (CMLM). The threshold for significant association was set to 1/n [n is the marker numbers, *P* ≤ 4.82 × 10^− 6^ or –log_10_(*P*) ≥ 5.32]. The LD decay rate of these 211 cultivated soybeans was 130 Kb [33].

### Haplotype analysis

Association analysis between SNPs and phenotypic value of shoot P concentration was calculated with Tassel 5.0 [47]. The LD level of pairwise SNPs was calculated by the HaploView 4.2 [48]. In addition, the haplotype block was defined by Gabriel et al. with “confidence intervals” algorithm [49].

### Induced expression analysis

In order to further confirm which of the two genes *Glyma.10 g018200* and *Glyma.10 g018800* identified by GWAS were related to P efficiency, hydroponic experiments were performed on two soybean accessions with different P efficiency. Seeds of two soybean accessions (Kefeng No. 1, P high efficiency; Nannong 1138–2, P low efficiency) were sterilized in Cl_2_ for 5 h, and germinated for 4 days in vermiculite. Uniform seedlings were selected and transferred to one-half Hoagland’s nutrient solution with 0.5 mM KH_2_PO_4_ for 3d. Roots were sampled at 15d after transferring to one-half Hoagland’s nutrient solution under +P and -P conditions, respectively. Three biological replicates per sample were frozen in liquid nitrogen and stored at − 80 °C for later isolation of total RNA.

### Bioinformatics analysis of the candidate gene

Using the full-length protein sequence of the candidate gene *Glyma.10 g018800* as the query sequence, BLAST searches were conducted in six species, *Arabidopsis* (*Arabidopsis thaliana*), soybean [*Glycine max* (L.) Merr.], common bean (*Phaseolus vulgaris*), alfalfa (*Medicago truncatula*), grape (*Vitis vinifera*) and rice (*Oryza sativa*), at Phytozome v12.1 website (https://phytozome.jgi.doe.gov/pz/portal.html). Eleven gene structures in six species were predicted by GSDS (http://gsds.gao-lab.org/index.php). The phylogenetic tree was drawn using MEGA 5.02 by the neighbor-joining method with bootstrap probabilities based on 1000 replicates. Nucleic acid sequence and amino acid sequence alignment was done with DNAman software. Prediction of gene conserved domains was performed online using NCBI (https://www.ncbi.nlm.nih.gov/Structure/cdd/wrpsb.cgi). The molecular weight and pI of candidate gene *GmSPX-RING1* was predicted by ExPASY (https://web.expasy.org/compute_pi/). Sequence analysis was performed using online program PLACE (http://www.dna.affrc.go.jp/PLACE/).

### Tissue expression of *GmSPX-RING1*

To analyze the expression pattern of *GmSPX-RING1* in different tissues of soybean, the roots, stems, leaves, flowers, pods and seeds of Nannong 1138–2 were sampled, and each tissue included three biological replicates. The sampling time of roots, stems and leaves was V4 stage of soybean vegetative growth, the sampling time of flower was R2 stage of soybean reproductive growth, and the sampling time of pods and seeds was 45 days after flowering.

### Subcellular localization

The coding sequence (without terminate codon) of *GmSPX-RING1* was amplified from cDNA of Nannong 1138–2, and cloned into pSuper1300 vector with GFP. The recombinant plasmid *GmSPX-RING1*-pSuper1300 and empty control were expressed in tobacco (*Nicotiana benthamiana*) leaves by *agrobacterium*-mediated infiltration (strain EHA105) as described previously [50]. The GFP fluorescence of tobacco leaves was imaged 2–3 d after infiltration using a microscope (Zeiss, LSM780). The primers used were listed in Additional file 11: Table S4.

### Soybean hairy root transformation

The coding sequence of *GmSPX-RING1* was cloned from cDNA of Nannong 1138–2, a P low efficiency soybean accession, and the verified PCR product was cloned into pMDC83 with the CaMV 35S promoter using double restriction enzyme digestion and recombination to obtain *GmSPX-RING1* overexpression vector (*GmSPX-RING1*-OE). A specific 405-bp fragment of *GmSPX-RING1* was amplified from *GmSPX-RING1*-OE, which cloned into pB7GWIWG2 (II) vector to obtain RNA interference of *GmSPX-RING1* vector (*GmSPX-RING1*-RNAi) using Gateway technology with the Clonase II Kit (Invitrogen CA, USA). The coding sequences of *GmSPX-RING1* in three soybean accessions, Jack, Kefeng No. 1 and Nannong 1138–2, were amplified and found to have only one SNP (C/A) with no difference in amino acid sequence i.e., is a synonymous mutation (Additional file 12: Figure S8). The primers used were listed in Additional file 11: Table S4.

*GmSPX-RING1*-OE, *GmSPX-RING1*-RNAi and their respective empty vectors (Control 1 and Control 2) were independently transformed into *Agrobacterium rhizogenes* strain K599 for soybean hairy root transformation. Jack is a soybean accession with high transformation efficiency. Soybean hairy root transformation was conducted using Jack as described previously [51]. Five-day-old seedlings were injected with K599, including *GmSPX-RING1*-OE, *GmSPX-RING1*-RNAi and their corresponding empty plasmids. The photoperiod and temperature were consistent with the hydroponic conditions of the natural population, but soybean hairy root required high humidity about 2–3 weeks. When the soybean hairy roots were about 2–10 cm in length, the primary roots were cut off. Then, soybean lines without primary roots after injected with *GmSPX-RING1*-OE, *GmSPX-RING1*-RNAi or its corresponding empty plasmid were transferred to +P or -P nutrient solution to culture for 15 days, each of which had 10–14 lines. Both pMDC83 and pB7GWIWG2 (II) vectors contained independently expressed green fluorescent protein (GFP). The GFP was observed by Luyor 3415 RG excitation source to screen soybean positive hairy root. After positive screening, there were fewer hairy roots. In order to ensure sufficient samples for further experiments, two soybean hairy root lines were combined into a biological replicate. Screened transgenic positive hairy roots were used for the measurement of *GmSPX-RING1* expression and P concentration.

### Detection of gene expression levels by qRT-PCR

The total RNA was isolated using RNAsimple Total RNA Kit (DP419, Tiangen, Beijing, China). The first strand of cDNA was synthesized using PrimeScript™ RT reagent Kit with gDNA Eraser (Perfect Real Time) (RR047A, Takara, Shanghai, China). qRT-PCR was conducted with the ABI 7500 system (Applied Biosystems, Foster City, CA, USA). The 20 μL reaction system consists of 10 μL 2 × RealUniversal PreMix (FP201, Tiangen, Beijing, China), 50 ng first-strand cDNAs and 0.6 μL each of 10 μM gene-specific primers. Data was analyzed using ABI 7500 system Sequence Detection System (SDS) software v.1.4. The relative expression was calculated as 2^-ΔΔCt^, ΔΔCt = (C_T, Target_-C_T, Tubulin_) _genotype_-(C_T, Target_-C_T, Tubulin_) _calibrator_ [52]. Tubulin (GenBank accession number: AY907703) was used as a control. The primers used were listed in Additional file 11: Table S4.

## Supplementary information

**Additional file 1: Figure S1.** Box plot of three P-efficiency related traits in natural population in two independent hydroponic experiments. SDWP: shoot dry weight under +P condition, SDWM: shoot dry weight under -P condition, SDWR: the ratio of shoot dry weight under -P condition to +P condition; SPP: shoot P concentration under +P condition, SPM: shoot P concentration under -P condition, SPR: the ratio of shoot P concentration under -P condition to +P condition; SPAP: shoot P accumulation under +P condition, SPAM: shoot P accumulation under -P condition, SPAR: the ratio of shoot P accumulation under -P condition to +P condition. E1/E2: first/second independent hydroponic culture.

**Additional file 2: Table S1.** Correlation coefficients of three P-efficiency traits in natural population under two P conditions. SDW: shoot dry weight; SP: shoot P concentration; SPA: shoot P accumulation. The correlation coefficients of natural populations under +P (0.5 mM KH_2_PO_4_) and -P (0.005 mM KH_2_PO_4_) conditions were listed in the upper right and lower left of the table, respectively. ns: not significant; *, **and *** significant at 0.05, 0.01 and 0.001 probability levels, respectively.

**Additional file 3: Figure S2.** Quantile-quantile (QQ) plots of three P efficiency related traits. SDWP: shoot dry weight under +P condition, SDWM: shoot dry weight under -P condition; SPP: shoot P concentration under +P condition, SPM: shoot P concentration under -P condition; SPAP: shoot P accumulation under +P condition, SPAM: shoot P accumulation under -P condition. E1/E2: first/second independent hydroponic culture. QQ plot of the same P efficiency traits in E1, E2 and mean of E1 and E2 were all in the same figure, dark green line represented four P-efficiency traits in E1, darkblue line represented four P-efficiency traits in E2, and green line represented four P-efficiency traits in mean of E1 and E2.

**Additional file 4: Figure S3.** Manhattan and QQ plots of relative values of two P efficiency related traits. SPR: the ratio of shoot P concentration under -P condition to +P condition; SPAR: the ratio of shoot P accumulation under -P condition to +P condition. The red line indicated the significance threshold (−log _10_ (*P*) =5.32).

**Additional file 5: Table S2.** Details of SNPs significantly associated with two P efficiency-related traits in only one hydroponic experiments. SDWP: shoot dry weight under +P condition, SDWM: shoot dry weight under -P condition, SDWR: the ratio of shoot dry weight under -P condition to +P condition; SPP: shoot P concentration under +P condition, SPM: shoot P concentration under -P condition, SPR: the ratio of shoot P concentration under -P condition to +P condition; SPAP: shoot P accumulation under +P condition, SPAM: shoot P accumulation under -P condition, SPAR: the ratio of shoot P accumulation under -P condition to +P condition. Chr.: chromosome; *P* value: significant at *P* ≤ 4.82 × 10^− 6^; *R*^*2*^: percentage of phenotypic variation explained by the SNP; Exp.: experiment; E1/E2: first/second independent hydroponic culture. Mean: average of the two experiments.

**Additional file 6: Figure S4.** Amino acid sequence alignment of homologous genes of *Glyma.10 g018800* and conserved domain analysis of *Glyma.10 g018800*. (a) Amino acid sequence alignment of *Glyma.10 g018800* in *Arabidopsis*, rice and soybean. (b) Conserved domain analysis of *Glyma.10 g018800*. Red line (2–138) stands for SPX domain, and green line (210–261) stands for RING domain.

**Additional file 7: Figure S5.** Expression pattern of *GmSPX-RING1* in different tissues of soybean. Data were the mean values of biological replicates mean ± standard deviation (SD) (*n* = 3). Statistical significance was detected by a two-tailed t-test. * and ** significant at 0.05 and 0.01 probability levels, respectively.

**Additional file 8: Figure S6.** Positive screening of soybean transgenic hairy roots by green fluorescence. *GmSPX-RING1*-OE: soybean transgenic hairy roots with *GmSPX-RING1* overexpression vector, Control 1: soybean transgenic hairy roots with overexpression empty vector; *GmSPX-RING1*-RNAi: soybean transgenic hairy roots with RNA interference of *GmSPX-RING1* vector, Control 2: soybean transgenic hairy roots with RNA interference empty vector. Negative Control: normal soybean roots without genetic transformation.

**Additional file 9: Figure S7.** Fresh weight of *GmSPX-RING1* transgenic hairy roots. (a) Fresh weight of *GmSPX-RING1*-OE transgenic hairy roots and Control 1 under +P and -P conditions. (b) Fresh weight of *GmSPX-RING1*-RNAi transgenic hairy roots and Control 2 under +P and -P conditions. *GmSPX-RING1*-OE: soybean transgenic hairy roots with *GmSPX-RING1* overexpression vector, Control 1: soybean transgenic hairy roots with overexpression empty vector; *GmSPX-RING1*-RNAi: soybean transgenic hairy roots with RNA interference of *GmSPX-RING1* vector, Control 2: soybean transgenic hairy roots with RNA interference empty vector. Data were the mean values of biological replicates mean ± standard deviation (SD) (*n* = 3). Statistical significance was detected by a two-tailed t-test. * and ** significant at 0.05 and 0.01 probability levels, respectively.

**Additional file 10: Table S3.** Geographic origins of 211 soybean accessions.

**Additional file 11: Table S4.** Primers used in this study.

**Additional file 12: Figure S8.** The coding sequence and amino acid sequence alignment of *GmSPX-RING1* in three soybean accessions. (a) Nucleic acid sequence of *GmSPX-RING1* in Jack, Kefeng No. 1 and Nannong 1138–2. (b) Amino acid sequence of *GmSPX-RING1* in Jack, Kefeng No. 1 and Nannong 1138–2.

## Data Availability

All data supporting the conclusions of this article is included within this article and its additional files.

## Abbreviations

SDW: Shoot dry weight
SDWP: Shoot dry weight under +P condition
SDWM: Shoot dry weight under -P condition
SDWR: The ratio of shoot dry weight under -P condition to +P condition
SP: Shoot P concentration
SPP: Shoot P concentration under +P condition
SPM: Shoot P concentration under -P condition
SPR: The ratio of shoot P concentration under -P condition to +P condition
SPA: Shoot P accumulation
SPAP: Shoot P accumulation under +P condition
SPAM: Shoot P accumulation under -P condition
SPAR: The ratio of shoot P accumulation under -P condition to +P condition
GWAS: Genome-wide association analysis
SNP: Single nucleotide polymorphism
qRT-PCR: Real-time quantitative polymerase chain reaction
NCBI: National Center for Biotechnology Information
GFP: Green Fluorescent protein
CaMV: Cauliflower mosaic virus
