# Supplementary material for: Identification of loci and candidate gene GmSPX-RING1 responsible for phosphorus efficiency in soybean via genome-wide association analysis
Source: BMC Genomics. 2020 Oct 19;21:725. doi: 10.1186/s12864-020-07143-3 (PMC7574279; doi:10.1186/s12864-020-07143-3)
Supplement: Supplementary file 1 — Additional file 1: Figure S1. Box plot of three P-efficiency related traits in natural population in two independent hydroponic experiments. SDWP: shoot dry weight under +P condition, SDWM: shoot dry weight under -P condition, SDWR: the ratio of shoot dry weight under -P condition to +P condition; SPP: shoot P concentration under +P condition, SPM: shoot P concentration under -P condition, SPR: the ratio of shoot P concentration under -P condition to +P condition; SPAP: shoot P accumulation under +P condition, SPAM: shoot P accumulation under -P condition, SPAR: the ratio of shoot P accumulation under -P condition to +P condition. E1/E2: first/second independent hydroponic culture. [file 12864_2020_7143_MOESM1_ESM.docx]

**
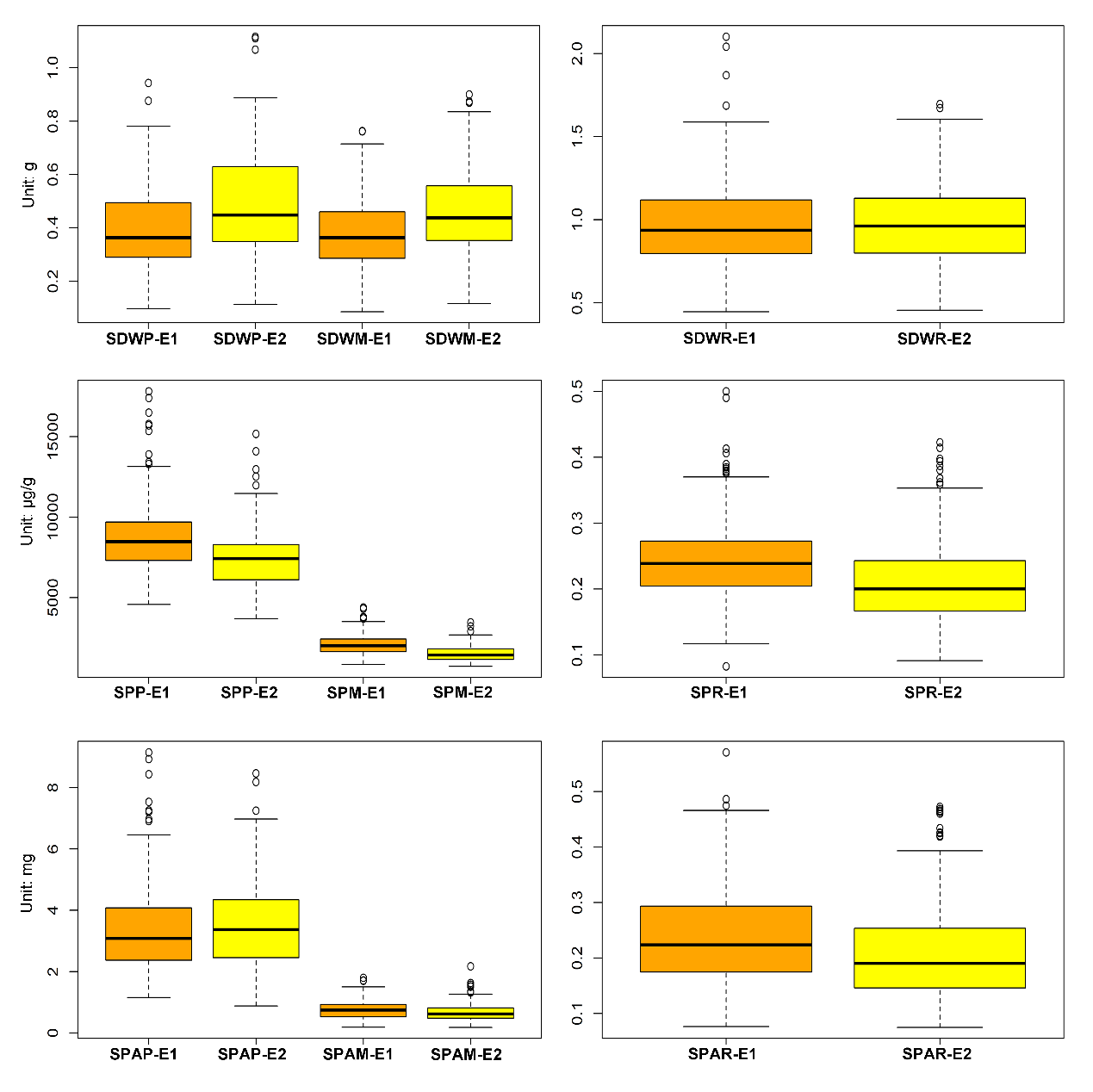
Additional file 1: Figure S1. Box plot of three P**-**efficiency related traits in natural population in two independent hydroponic experiments.**

SDWP: shoot dry weight under +P condition, SDWM: shoot dry weight under -P condition, SDWR: the ratio of shoot dry weight under -P condition to +P condition; SPP: shoot P concentration under +P condition, SPM: shoot P concentration under -P condition, SPR: the ratio of shoot P concentration under -P condition to +P condition; SPAP: shoot P accumulation under +P condition, SPAM: shoot P accumulation under -P condition, SPAR: the ratio of shoot P accumulation under -P condition to +P condition. E1/E2: first/second independent hydroponic culture.
