# Supplementary material for: Identification of loci and candidate gene GmSPX-RING1 responsible for phosphorus efficiency in soybean via genome-wide association analysis
Source: BMC Genomics. 2020 Oct 19;21:725. doi: 10.1186/s12864-020-07143-3 (PMC7574279; doi:10.1186/s12864-020-07143-3)
Supplement: Supplementary file 3 — Additional file 3: Figure S2. Quantile-quantile (QQ) plots of three P efficiency related traits. SDWP: shoot dry weight under +P condition, SDWM: shoot dry weight under -P condition; SPP: shoot P concentration under +P condition, SPM: shoot P concentration under -P condition; SPAP: shoot P accumulation under +P condition, SPAM: shoot P accumulation under -P condition. E1/E2: first/second independent hydroponic culture. QQ plot of the same P efficiency traits in E1, E2 and mean of E1 and E2 were all in the same figure, dark green line represented four P-efficiency traits in E1, darkblue line represented four P-efficiency traits in E2, and green line represented four P-efficiency traits in mean of E1 and E2. [file 12864_2020_7143_MOESM3_ESM.docx]

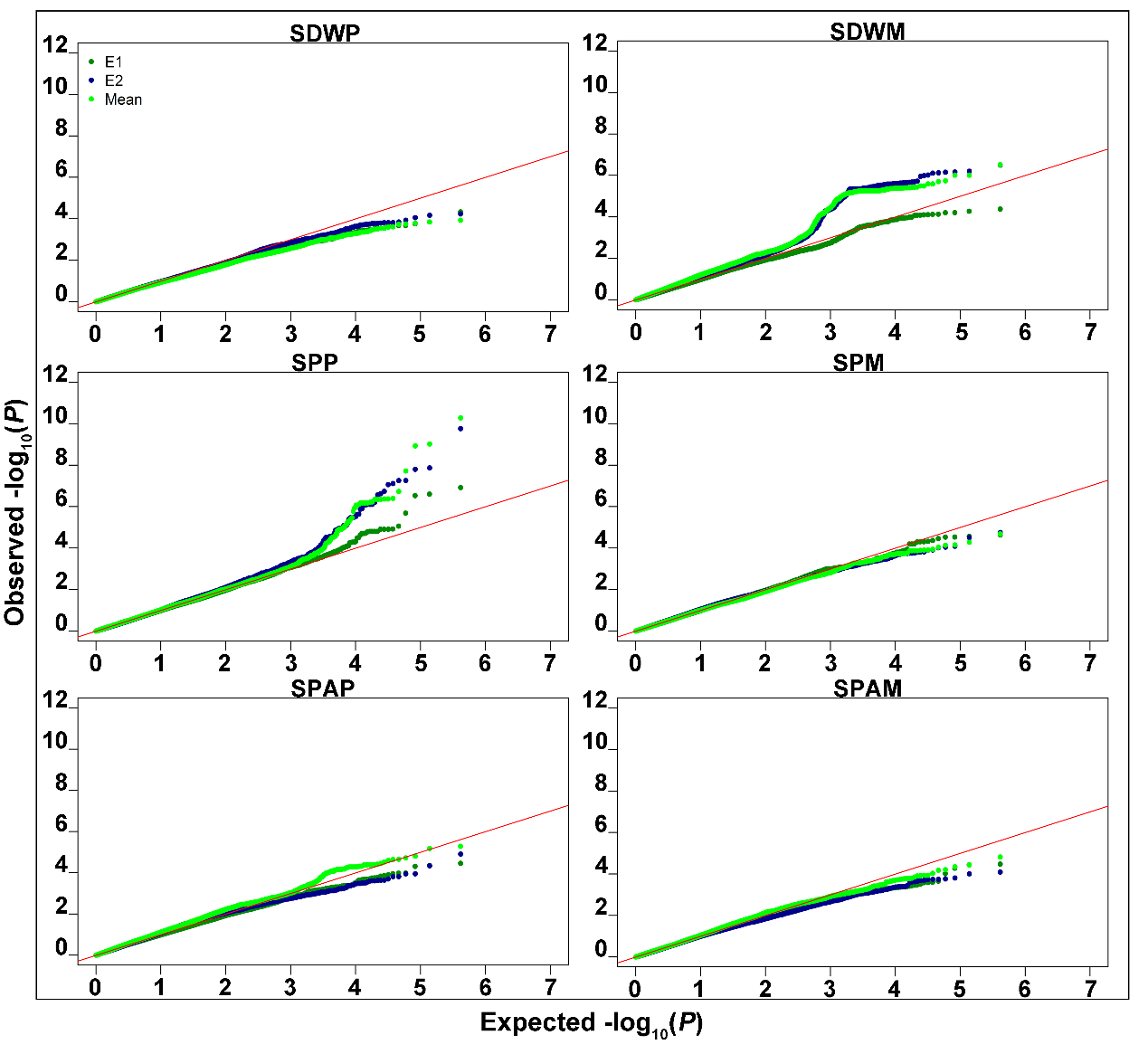


**Additional file 3: Figure S2. Quantile-quantile (QQ) plots of three P efficiency related traits.**

SDWP: shoot dry weight under +P condition, SDWM: shoot dry weight under -P condition; SPP: shoot P concentration under +P condition, SPM: shoot P concentration under -P condition; SPAP: shoot P accumulation under +P condition, SPAM: shoot P accumulation under -P condition. E1/E2: first/second independent hydroponic culture. QQ plot of the same P efficiency traits in E1, E2 and mean of E1 and E2 were all in the same figure, dark green line represented four P-efficiency traits in E1, darkblue line represented four P-efficiency traits in E2, and green line represented four P-efficiency traits in mean of E1 and E2.
