# Supplementary material for: Identification of loci and candidate gene GmSPX-RING1 responsible for phosphorus efficiency in soybean via genome-wide association analysis
Source: BMC Genomics. 2020 Oct 19;21:725. doi: 10.1186/s12864-020-07143-3 (PMC7574279; doi:10.1186/s12864-020-07143-3)
Supplement: Supplementary file 4 — Additional file 4: Figure S3. Manhattan and QQ plots of relative values of two P efficiency related traits. SPR: the ratio of shoot P concentration under -P condition to +P condition; SPAR: the ratio of shoot P accumulation under -P condition to +P condition. The red line indicated the significance threshold (−log 10 (P) =5.32). [file 12864_2020_7143_MOESM4_ESM.docx]

**
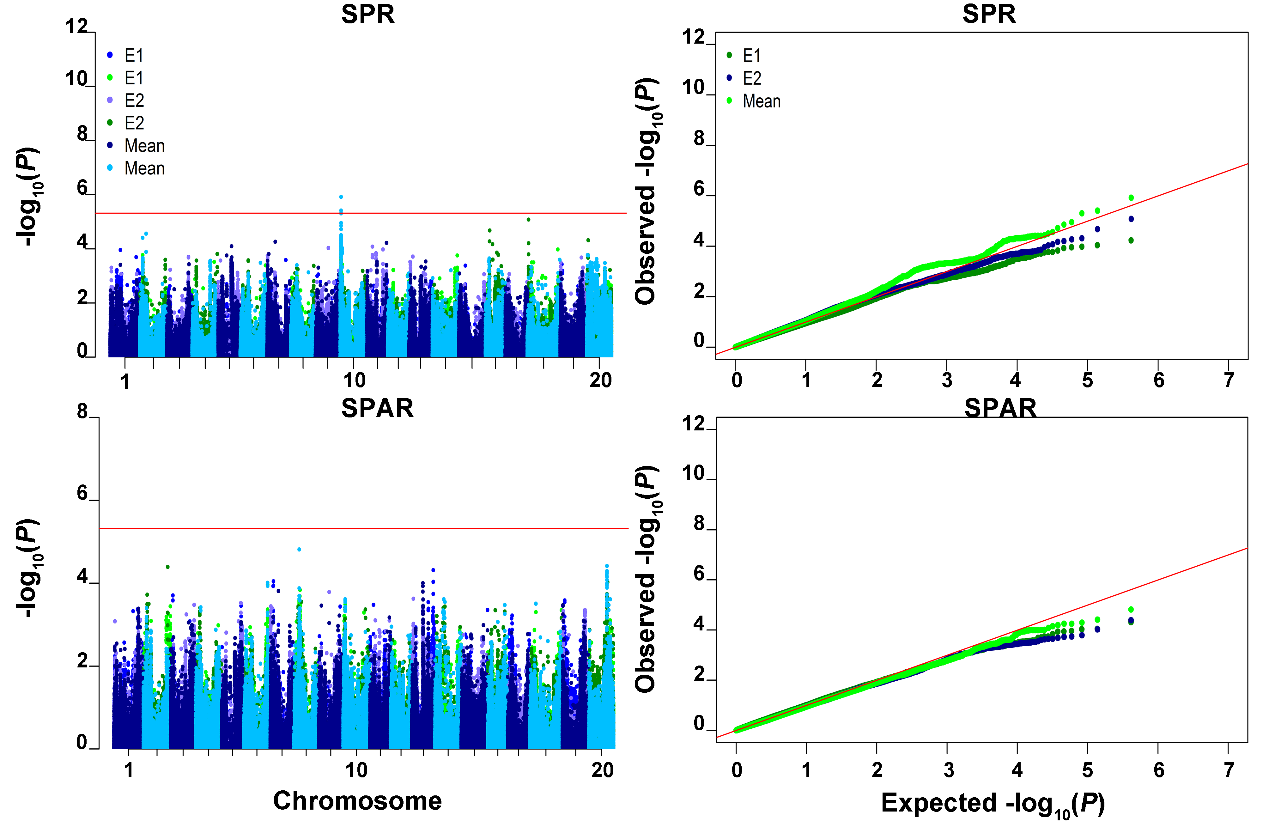
Additional file 4: Figure S3. Manhattan and QQ plots of relative values of two P efficiency related traits.**

SPR: the ratio of shoot P concentration under -P condition to +P condition; SPAR: the ratio of shoot P accumulation under -P condition to +P condition. The red line indicated the significance threshold (-log _10_ (*P*) =5.32).
