# Supplementary material for: Identification of loci and candidate gene GmSPX-RING1 responsible for phosphorus efficiency in soybean via genome-wide association analysis
Source: BMC Genomics. 2020 Oct 19;21:725. doi: 10.1186/s12864-020-07143-3 (PMC7574279; doi:10.1186/s12864-020-07143-3)
Supplement: Supplementary file 6 — Additional file 6: Figure S4. Amino acid sequence alignment of homologous genes of Glyma.10 g018800 and conserved domain analysis of Glyma.10 g018800. (a) Amino acid sequence alignment of Glyma.10 g018800 in Arabidopsis, rice and soybean. (b) Conserved domain analysis of Glyma.10 g018800. Red line (2–138) stands for SPX domain, and green line (210–261) stands for RING domain. [file 12864_2020_7143_MOESM6_ESM.docx]

**
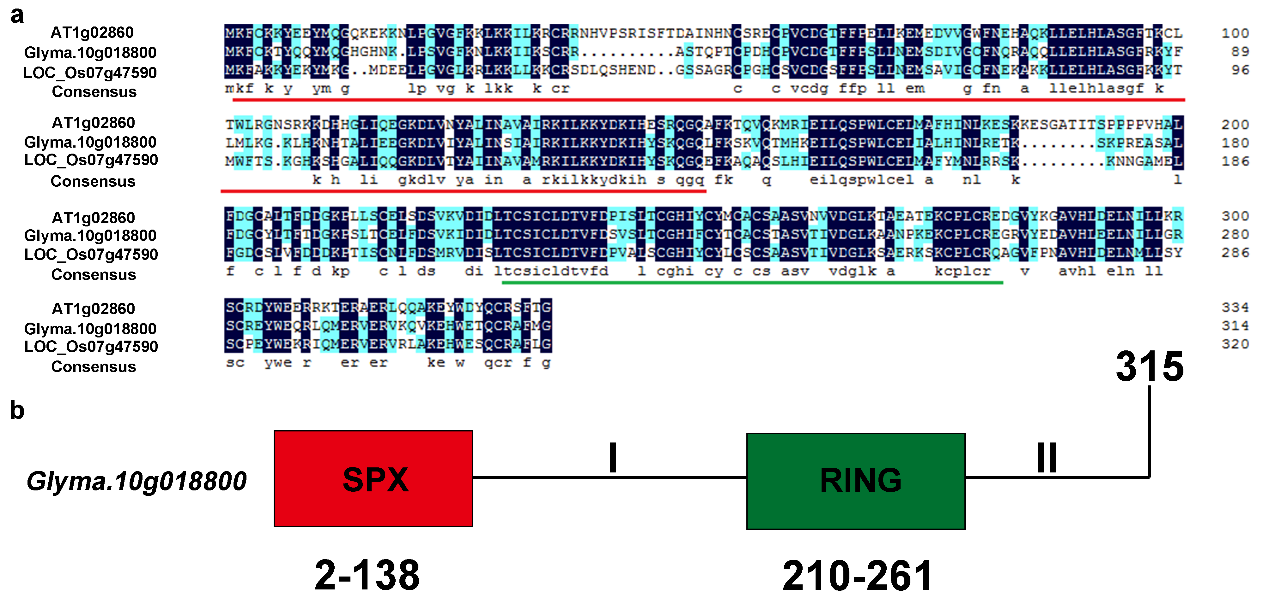
Additional file 6: Figure S4. Amino acid sequence alignment of homologous genes of *Glyma.10g018800* and conserved domain analysis of *Glyma.10g018800*.**

(a) Amino acid sequence alignment of *Glyma.10g018800* in *Arabidopsis*, rice and soybean. (b) Conserved domain analysis of *Glyma.10g018800*. Red line (2-138) stands for SPX domain, and green line (210-261) stands for RING domain.
