# Supplementary material for: Identification of loci and candidate gene GmSPX-RING1 responsible for phosphorus efficiency in soybean via genome-wide association analysis
Source: BMC Genomics. 2020 Oct 19;21:725. doi: 10.1186/s12864-020-07143-3 (PMC7574279; doi:10.1186/s12864-020-07143-3)
Supplement: Supplementary file 7 — Additional file 7: Figure S5. Expression pattern of GmSPX-RING1 in different tissues of soybean. Data were the mean values of biological replicates mean ± standard deviation (SD) (n = 3). Statistical significance was detected by a two-tailed t-test. * and ** significant at 0.05 and 0.01 probability levels, respectively. [file 12864_2020_7143_MOESM7_ESM.docx]

**
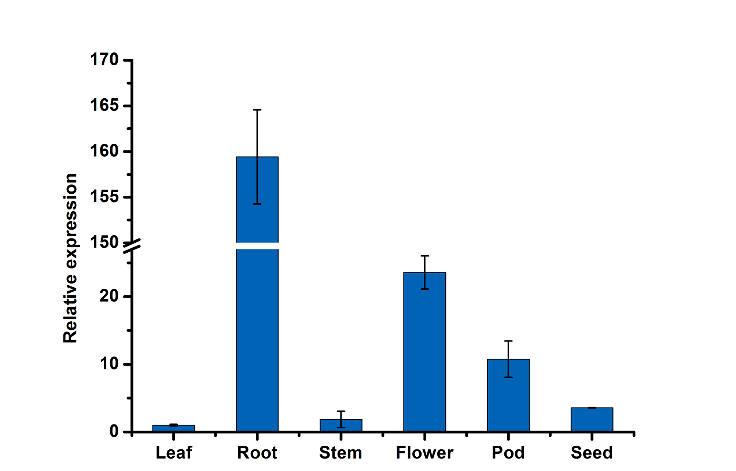
Additional file 7: Figure S5. Expression pattern of *GmSPX-RING1* in different tissues of soybean.**

Data are the mean values of biological replicates mean ± standard deviation (SD) (n=3). Statistical significance was detected by a two-tailed t-test. * and ** significant at 0.05 and 0.01 probability levels, respectively.
