# Supplementary material for: Identification of loci and candidate gene GmSPX-RING1 responsible for phosphorus efficiency in soybean via genome-wide association analysis
Source: BMC Genomics. 2020 Oct 19;21:725. doi: 10.1186/s12864-020-07143-3 (PMC7574279; doi:10.1186/s12864-020-07143-3)
Supplement: Supplementary file 8 — Additional file 8: Figure S6. Positive screening of soybean transgenic hairy roots by green fluorescence. GmSPX-RING1-OE: soybean transgenic hairy roots with GmSPX-RING1 overexpression vector, Control 1: soybean transgenic hairy roots with overexpression empty vector; GmSPX-RING1-RNAi: soybean transgenic hairy roots with RNA interference of GmSPX-RING1 vector, Control 2: soybean transgenic hairy roots with RNA interference empty vector. Negative Control: normal soybean roots without genetic transformation. [file 12864_2020_7143_MOESM8_ESM.docx]

**
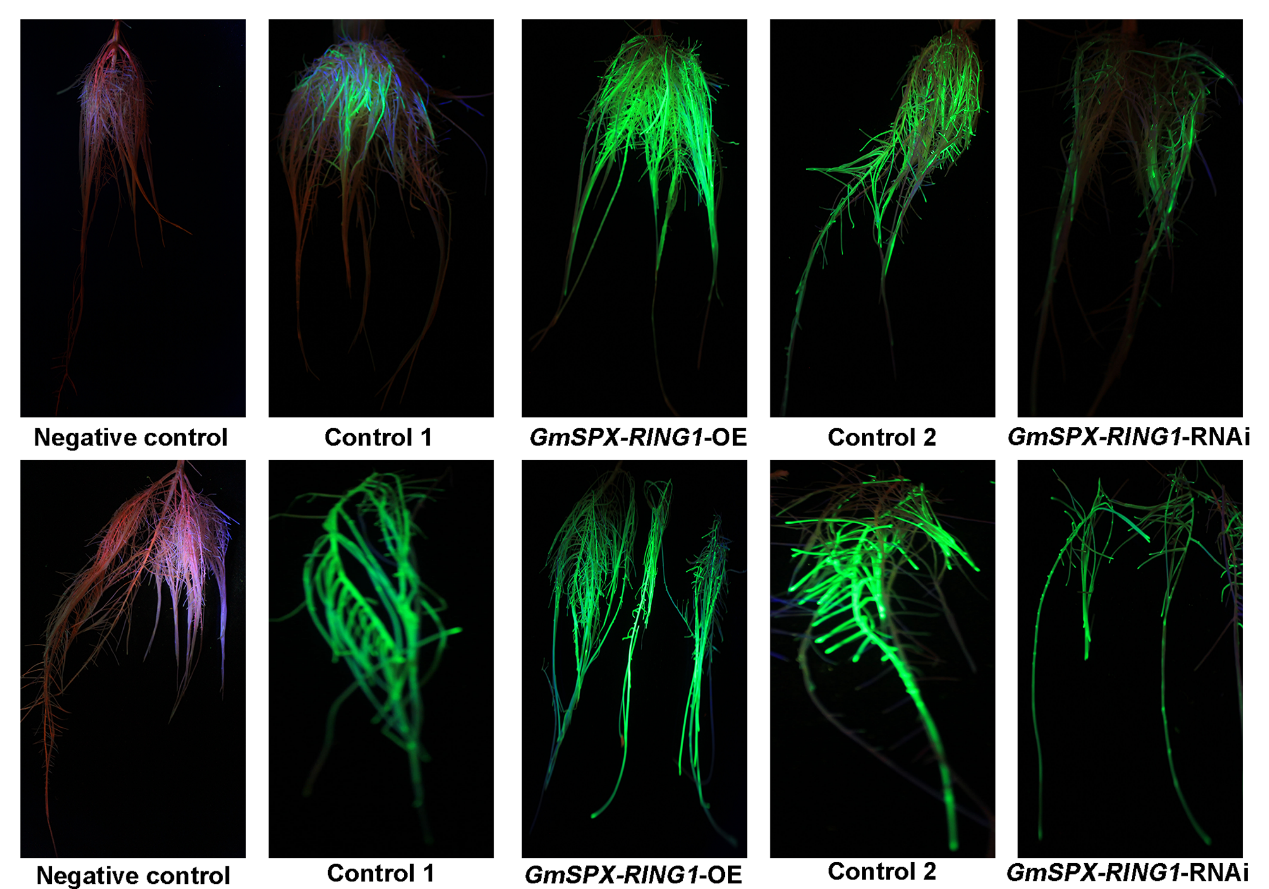
Additional file 8: Figure S6. Positive screening of soybean transgenic hairy roots by green fluorescence.**

*GmSPX-RING1*-OE: soybean transgenic hairy roots with *GmSPX-RING1* overexpression vector, Control 1: soybean transgenic hairy roots with overexpression empty vector; *GmSPX-RING1*-RNAi: soybean transgenic hairy roots with RNA interference of *GmSPX-RING1* vector, Control 2: soybean transgenic hairy roots with RNA interference empty vector. Negative Control: normal soybean roots without genetic transformation.
