# Supplementary material for: Identification of loci and candidate gene GmSPX-RING1 responsible for phosphorus efficiency in soybean via genome-wide association analysis
Source: BMC Genomics. 2020 Oct 19;21:725. doi: 10.1186/s12864-020-07143-3 (PMC7574279; doi:10.1186/s12864-020-07143-3)
Supplement: Supplementary file 9 — Additional file 9: Figure S7. Fresh weight of GmSPX-RING1 transgenic hairy roots. (a) Fresh weight of GmSPX-RING1-OE transgenic hairy roots and Control 1 under +P and -P conditions. (b) Fresh weight of GmSPX-RING1-RNAi transgenic hairy roots and Control 2 under +P and -P conditions. GmSPX-RING1-OE: soybean transgenic hairy roots with GmSPX-RING1 overexpression vector, Control 1: soybean transgenic hairy roots with overexpression empty vector; GmSPX-RING1-RNAi: soybean transgenic hairy roots with RNA interference of GmSPX-RING1 vector, Control 2: soybean transgenic hairy roots with RNA interference empty vector. Data were the mean values of biological replicates mean ± standard deviation (SD) (n = 3). Statistical significance was detected by a two-tailed t-test. * and ** significant at 0.05 and 0.01 probability levels, respectively. [file 12864_2020_7143_MOESM9_ESM.docx]

**
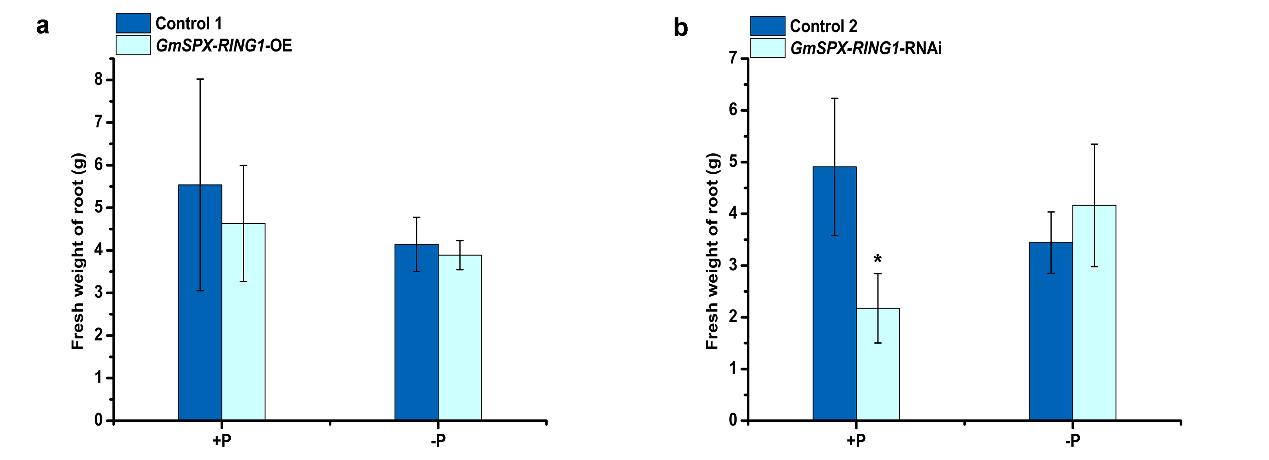
Additional file 9: Figure S7. Fresh weight of *GmSPX-RING1* transgenic hairy roots.**

(a) Fresh weight of *GmSPX-RING1*-OE transgenic hairy roots and Control 1 under +P and -P conditions. (b) Fresh weight of *GmSPX-RING1*-RNAi transgenic hairy roots and Control 2 under +P and -P conditions. *GmSPX-RING1*-OE: soybean transgenic hairy roots with *GmSPX-RING1* overexpression vector, Control 1: soybean transgenic hairy roots with overexpression empty vector; *GmSPX-RING1*-RNAi: soybean transgenic hairy roots with RNA interference of *GmSPX-RING1* vector, Control 2: soybean transgenic hairy roots with RNA interference empty vector. Data are the mean values of biological replicates mean ± standard deviation (SD) (n=3). Statistical significance was detected by a two-tailed t-test. * and ** significant at 0.05 and 0.01 probability levels, respectively.
