# Supplementary material for: Identification of loci and candidate gene GmSPX-RING1 responsible for phosphorus efficiency in soybean via genome-wide association analysis
Source: BMC Genomics. 2020 Oct 19;21:725. doi: 10.1186/s12864-020-07143-3 (PMC7574279; doi:10.1186/s12864-020-07143-3)
Supplement: Supplementary file 12 — Additional file 12: Figure S8. The coding sequence and amino acid sequence alignment of GmSPX-RING1 in three soybean accessions. (a) Nucleic acid sequence of GmSPX-RING1 in Jack, Kefeng No. 1 and Nannong 1138–2. (b) Amino acid sequence of GmSPX-RING1 in Jack, Kefeng No. 1 and Nannong 1138–2. [file 12864_2020_7143_MOESM12_ESM.docx]

**
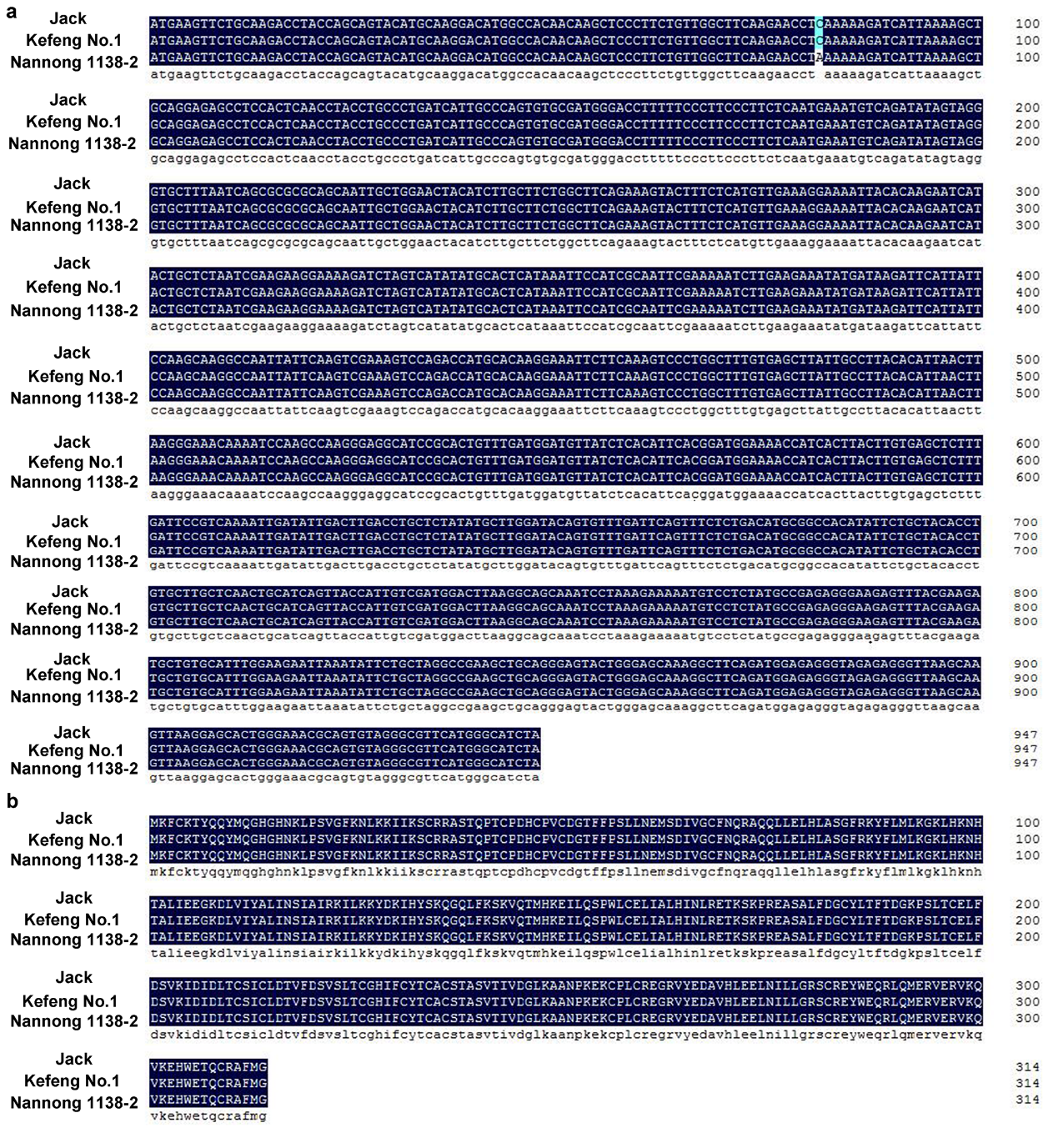
Additional file 12: Figure S8. The coding sequence and amino acid sequence alignment of *GmSPX-RING1* in three soybean accessions.**

(a) Nucleic acid sequence of *GmSPX-RING1* in Jack, Kefeng No. 1 and Nannong 1138-2. (b) Amino acid sequence of *GmSPX-RING1* in Jack, Kefeng No. 1 and Nannong 1138-2.
